# Compartmentalization and Functionality of Nuclear Disorder: Intrinsic Disorder and Protein-Protein Interactions in Intra-Nuclear Compartments

**DOI:** 10.3390/ijms17010024

**Published:** 2015-12-25

**Authors:** Fanchi Meng, Insung Na, Lukasz Kurgan, Vladimir N. Uversky

**Affiliations:** 1 Department of Electrical and Computer Engineering, University of Alberta, Edmonton, AB T6G 2V4, Canada; fanchi@ualberta.ca; 2 Department of Molecular Medicine, Morsani College of Medicine, University of South Florida, Tampa, FL 33612, USA; ina2@health.usf.edu; 3 Department of Computer Science, Virginia Commonwealth University, Richmond, VA 23219, USA; 4 University of South Florida Health Byrd Alzheimer’s Research Institute, Morsani College of Medicine, University of South Florida, Tampa, FL 33612, USA; 5 Institute for Biological Instrumentation, Russian Academy of Sciences, Pushchino, Moscow Region 142292, Russian; 6 Biology Department, Faculty of Science, King Abdulaziz University, P.O. Box 80203, Jeddah 21589, Saudi Arabia; 7 Laboratory of Structural Dynamics, Stability and Folding of Proteins, Institute of Cytology, Russian Academy of Sciences, Saint Petersburg 194064, Russian

**Keywords:** intrinsically disordered protein, cell nucleus, membrane-less organelles, intra-nuclear compartments, DNA-binding protein, RNA-binding protein

## Abstract

The cell nucleus contains a number of membrane-less organelles or intra-nuclear compartments. These compartments are dynamic structures representing liquid-droplet phases which are only slightly denser than the bulk intra-nuclear fluid. They possess different functions, have diverse morphologies, and are typically composed of RNA (or, in some cases, DNA) and proteins. We analyzed 3005 mouse proteins localized in specific intra-nuclear organelles, such as nucleolus, chromatin, Cajal bodies, nuclear speckles, promyelocytic leukemia (PML) nuclear bodies, nuclear lamina, nuclear pores, and perinuclear compartment and compared them with ~29,863 non-nuclear proteins from mouse proteome. Our analysis revealed that intrinsic disorder is enriched in the majority of intra-nuclear compartments, except for the nuclear pore and lamina. These compartments are depleted in proteins that lack disordered domains and enriched in proteins that have multiple disordered domains. Moonlighting proteins found in multiple intra-nuclear compartments are more likely to have multiple disordered domains. Protein-protein interaction networks in the intra-nuclear compartments are denser and include more hubs compared to the non-nuclear proteins. Hubs in the intra-nuclear compartments (except for the nuclear pore) are enriched in disorder compared with non-nuclear hubs and non-nuclear proteins. Therefore, our work provides support to the idea of the functional importance of intrinsic disorder in the cell nucleus and shows that many proteins associated with sub-nuclear organelles in nuclei of mouse cells are enriched in disorder. This high level of disorder in the mouse nuclear proteins defines their ability to serve as very promiscuous binders, possessing both large quantities of potential disorder-based interaction sites and the ability of a single such site to be involved in a large number of interactions.

## 1. Introduction

The nucleus is a double membrane-bound organelle, which is the most characteristic and important component of any eukaryotic cell, serving as the “container” of genetic information that is responsible for the storage of hereditary material (in chromosomes and genes), storage of proteins and RNA (specifically in the nucleolus), exchange of hereditary molecules (DNA and RNA), production of ribosomes, and coordination of the gene expression [1,2,3,4]. Although the nucleus typically takes ~6% of the total cellular volume, in some cell types, nucleus occupies 30%–50% of the cellular volume. The nucleus is a highly dynamic organelle, and its morphology, size, and shape are tightly regulated and are noticeably changed during the cell cycle. Development of several diseases, such as cancer, is correlated with the altered nuclear morphology [4].

It is recognized now that the cell nucleus is a non-homogeneous entity filled with a transparent, semi-solid, granular substance known as nucleoplasm, nuclear sap or karyolymph, that has a complex architecture and contains several sub-nuclear structures alias nuclear domains, or sub-nuclear organelles, or nuclear compartments, or sub-nuclear bodies [5]. These sub-nuclear compartments are temporary and are classified as highly dynamic, membrane-less organelles, which do not exist all the time, appear only during certain stages of the cell cycle, and are dynamically formed via recruitment of proteins, RNA and DNA. At the moment, little information is available on the highly dynamic nuclear environment, on the roles of various nuclear organelles, domains and compartments, and on the existence and functional roles of interactions between these organelles and compartments. Furthermore, the molecular mechanisms defining the ability of these nuclear domains to maintain their specific structures in the absence of membranes also remain mostly unknown [6]. Since they lack membranes, these organelles possess a highly dynamic nature, and their components are involved in direct contact with the surrounding nucleoplasm or cytoplasm [7,8]. These membrane-less compartments with their high level of internal dynamics are marginally denser than the bulk intra-nuclear/intra-cellular fluid, and are, therefore, considered as liquid-droplet phases of the nucleoplasm/cytoplasm [9,10,11,12,13,14].

Among these sub-nuclear organelles are Cajal bodies, chromatin, cleavage bodies, nuclear gems (Gemini of coiled bodies), nuclear pores, nuclear speckles, nucleolus, Oct1/PTF/transcription (OPT) domains, PcG bodies (sub-nuclear organelles containing polycomb group proteins), perinuclear compartment, promyelocytic leukemia (PML) nuclear bodies, SAM68 nuclear bodies, and a few others [15]. Several of these sub-nuclear structures (such as nucleolus, chromatin, Cajal bodies, nuclear speckles, PML bodies, nuclear lamina, nuclear pores, and perinuclear compartment) are subjects of the analysis conducted in our study, and therefore they are briefly introduced below.

Nucleolus is one of the most important sub-nuclear organelle which is clearly visible in the nucleoplasm as a large, dense, spherical structure with pronounced acidophilic character. The main function of the nucleolus is the biogenesis of ribosomal subunits, which, being synthesized, are taken to the cytoplasm for the translation of RNA. Therefore, the size of nucleolus is related to the ribosomal demands of the cell, where cells producing large quantities of proteins (and, thereby, requiring more ribosomes) have large nucleolus, and cells with no synthetic activities (such as spermatozoids or muscle cells) have smaller nucleolus or do not have nucleolus at all [16]. The typical nucleolus is composed of three regions: a fibrillar center containing DNA that is not actively transcribed, a dense fibrillar component containing RNA molecules which are actively transcribed, and a granular component containing maturing ribosomal precursor particles [17]. The nucleolar proteins represent the largest subset of nuclear proteins analyzed in this study.

Chromatin or chromatin fibers are thread-like, coiled, elongated structures made of DNA and proteins and found in the nucleoplasm. The primary functions ascribed to chromatin are: (i) DNA packaging into a smaller volume to fit in the cell; (ii) DNA reinforcement to allow mitosis; (iii) prevention of the DNA damage; and (iv) providing means to control DNA replication and gene expression [18]. Therefore, chromatin is a way of dynamic storage of long DNA molecules. In the eukaryotic nucleus, histone proteins participate in the hierarchical packaging of genomic DNA. Here, the (H3-H4)_2_ tetramer capped with two dimers of H2A-H2B constitute a core histone octamer, around which the 146 base pairs of DNA are wrapped in 1.7 super-helical turns to form the nucleosome, chromatin’s fundamental repeating unit [19,20,21]. The resulting “beads-on-a-string” fiber that has a diameter of 11 nm serves as the first level of organization for chromatin [20]. Meanwhile, the subsequent binding of the linker histone (H1 or H5) forms a more condensed 30-nm fiber, which generates the second structural level of DNA organization [21,22]. Multiple mechanisms ranging from histone modification to chromatin remodeling, to histone eviction, and to histone variant incorporation are involved in tight regulation of the chromatin structure dynamics [18]. Chromatin is unevenly distributed within the nucleus, being organized into the chromosome territories [15,23,24]. Nuclear speckles, which are also known as interchromatin granule clusters, are the storage sites for the pre-mRNA splicing factors that can be later recruited by RNA polymerase II transcription sites in the nucleoplasm. The cell nucleus contains 25–50 such speckles which are diffusely distributed throughout the nucleoplasm and that include a set of pre-mRNA splicing factors [25].

Nucleus typically has one to five Cajal bodies (CBs), which are roughly spherical 0.1–2.0 µM sub-nuclear structures. Although CBs are not easily detected in all cell types, they have very prominent appearance in the highly active cells such as cancer cells or neurons [26,27]. CBs are known as nucleolar accessory bodies or coiled bodies because of the presence of the coiled threads of the specific protein, coilin [26,27]. Since CBs are able to concentrate some of the components related to the several nuclear processes (e.g., they are involved in the assembly of U small nuclear ribonucleoproteins (snRNPs) needed for the formation of the spliceosome), these sub-organelles are assumed to increase efficiency of the related processes [27].

The average mammalian nucleus contains between 10 and 30 of promyelocytic leukemia (PML) bodies. These organelles with the diameter ranging from 0.3 to 1.0 µm are also known as ND10, Kr bodies, and PODs (PML oncogenic domains) [28]. PML bodies can be found in proximity of other nuclear organelles (e.g., Cajal bodies and nuclear gems). However, functional significance of this juxtaposition is unknown. Although the central core of the PML bodies are devoid of chromatin and RNA, the newly synthesized RNAs are found at their periphery [29]. On late-replicated satellite DNA, these sub-nuclear organelles are believed to re-establish the condensed heterochromatic state [30].

The nuclear envelope separates the content of the nucleus, which is the double membrane-enclosed cellular organelle, from the cytoplasm, and which is known to disappear during cell division. The nuclear envelope has two layers, an outer membrane and an inner membrane (also known as nuclear lamina). Each of these lipoproteinaceous layers is about 75 to 90 Å thick, with the space between theme being known as the perinuclear space [31]. Both these layers enable the exchange of ions between the nucleus and cytoplasm, and in some cell types (such as salivary glands) these two structures work as a barrier for the diffusion of substances and some ions, such as K^+^, Na^+^, and chloride Cl^−^ [32,33,34].

Nuclear pores are abundantly found in the nuclear envelope. The nuclear pore complex is a large, multichain proteinaceous machine (124 MDa in mammals), which is arranged hexagonally along the membrane, crosses the nuclear envelope and contains approximately 30 different protein components (nucleoporins), each in multiple copies [35]. On average, these pores cover 10% of the surface of the nucleus in mammalian cells. In some cases, the nuclear pore complex is additionally covered by a thin membrane which is potentially involved in selective permeability [35,36,37].

An irregularly shaped nuclear body known as the perinucleolar compartment (PNC) is found at the periphery of the nucleolus [38,39,40]. The length of PNC ranges from 0.25 to 4.0 µm, and this organelle contains several short RNAs and the heterogeneous nuclear ribonucleoprotein I, which is a polypyrimidine tract-binding protein [38].

It is of interest to point out that the membrane-less compartments are not unique for the nucleus, and several of the non-membrane bound organelles are found in the cytoplasm of eukaryotic cells. Often, these organelles (such as centrosomes [41], spindles [42], and stress granules [14], to name a few) are comprised of proteins and RNA [42].

Although these sub-nuclear organelles are rather different morphologically and have very different functions, they are typically composed of RNA (or, in some cases, DNA) and proteins. Recent studies revealed that in contrast to the classical protein structure paradigm stating that a protein must have a defined 3D-structure in order to be functional, many biologically active proteins with a wide spectrum of crucial functions are characterized by the lack of such specific tertiary structures [43,44,45,46,47,48,49]. Studies of different proteomes suggested that both intrinsically disordered proteins (IDPs) and hybrid proteins containing intrinsically disordered protein regions (IDPRs) as well as ordered domains are very prevalent in nature and that proteins from eukaryotes have more intrinsic disorder than proteins of bacteria or archaea [46,50,51,52,53,54,55,56]. Disorder-based functions are typically related to control pathways, signaling, and regulation [57,58,59,60,61], being therefore complementary to the catalytic and transport functions of ordered proteins [62,63,64,65]. Since among the disorder-specific functions are DNA- and RNA-interactions, and since almost all sub-nuclear organelles contain RNA- and DNA-binding proteins, our goal was to study the nuclear proteins of the mouse cell, to empirically evaluate their level of disorder, to investigate whether there is a connection between intrinsic disorder and the functional roles of these proteins in the cell nucleus, and, thereby, to better understand the role of the intrinsic disorder in the context of compartmentalization of the nucleus. We also empirically evaluated certain characteristics of protein-protein interaction (PPI) network, such as density and presence of highly connected protein hubs, in the considered intra-nuclear compartments and analyzed the relation between these characteristics and the intrinsic disorder.

The current work extends our earlier study on the analysis of disorder status of 185 human nuclear proteins [66]. Here, we use a much larger dataset (3005 *vs.* 185 nuclear proteins), apply multiple disorder prediction tools *vs.* a single tool to strengthen confidence in the estimation of the abundance of disorder, conduct analysis of PPIs and hubs, perform analysis of the proteins that are co-localized in multiple compartments, and provide richer set of measures to quantify abundance of disorder (proteins with multiple disordered domains and normalized content of disordered domains).

## 2. Results and Discussion

### 2.1. Intrinsic Disorder in the Annotated and Predicted Intra-Nuclear Localizations Is Equivalent

Table 1 lists datasets used in our study. First, we compared the disorder content (Figure S1) and other characteristics of the intrinsic disorder (Tables S1 and S2) across the experimentally annotated intra-nuclear localizations (NUCLEAR*_a_*), annotated and predicted intra-nuclear localizations (NUCLEAR*_ap_*), and the annotated and predicted intra-nuclear localizations that were mapped into the PPI network (PPI_NUCLEAR*ap*_). Figure S1 reveals that distributions of the disorder content are very similar between these three sets of annotated proteins.

**Table 1:** Datasets used in this study.

Dataset	Description	Number of Proteins
NNUCLEAR	Complete mouse proteome excluding the nuclear proteins.	29,863
NUCLEAR*_a_*	Nuclear mouse proteome with annotated intra-nuclear compartments.	1285
NUCLEAR*_ap_*	Nuclear mouse proteome with annotated and predicted intra-nuclear compartments.	3005
PPI	Mouse proteins included in the protein-protein interaction (PPI) network.	8206
PPI_NNUCLEAR_	Complete mouse proteome without nuclear proteins included in the PPI network (intersection of NNUCLEAR and PPI).	5687
PPI_NUCLEAR*ap*_	Nuclear mouse proteome with annotated and predicted intra-nuclear compartments included in the PPI network (intersection of NUCLEAR*_ap_* and PPI).	2519

The two supplementary Tables also show that the fraction of proteins with disordered domains and completely disordered proteins are similar. Therefore, in the subsequent analyses, we report our results on the larger combined set of annotated and predicted localization; the corresponding results on the smaller set of annotated localizations are given in the Supplement. Furthermore, this similarity also justifies our use of the datasets mapped into the PPI network to analyze the peculiarities of the protein-protein interactions in relation to the intrinsic disorder for the intra-nuclear compartments.

### 2.2. Intrinsic Disorder in Intra-Nuclear Compartments

**Figure 1:**
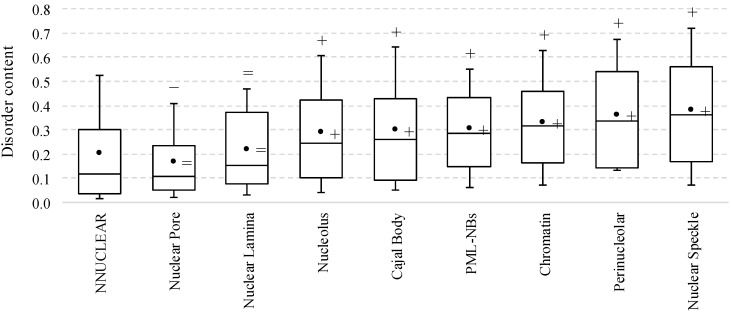
Disorder content of the non-nuclear proteins from the NNUCLEAR dataset and proteins in the considered intra-nuclear compartment from the NUCLEAR*_ap_* dataset. The box plots include the first quartile, median and third quartile while whiskers correspond to the 10th and 90th centiles of the disorder content of proteins in a given set. The black circle marker is the mean value of the disorder content. The significance of the differences in the median (mean) disorder content between proteins in a given compartment and non-nuclear proteins is annotated above the whiskers (right of the marker); + and − mean that the content of the nuclear proteins is significantly higher and lower (*p*-value < 0.01), respectively; “=” means that the difference is not significant (*p*-value ≥ 0.01). Disorder was annotated with the consensus of Espritz and IUPred.

Figure 1 compares the disorder content between the non-nuclear proteins and the nuclear proteins localized in intra-nuclear compartments (Figure S2 shows the corresponding results based solely on the experimental annotations of localization). Nuclear proteins in most of the compartments, except for nuclear pore and lamina, are significantly (*p*-value < 0.01) enriched in the disorder compared to the non-nuclear proteins. The median disorder content is higher by between 108% in the nucleolus (0.25 *vs.* 0.12 for the non-nuclear proteins) and 200% in the nuclear speckle (0.36 *vs.* 0.12). The lower disorder content in the nuclear pore could be explained by the highly structured composition of the nuclear pore complex [67]. Nuclear lamina has median content of 0.15 which is slightly higher (*p*-value = 0.07) than the overall content of 0.12 for the non-nuclear proteins.

**Figure 2:**
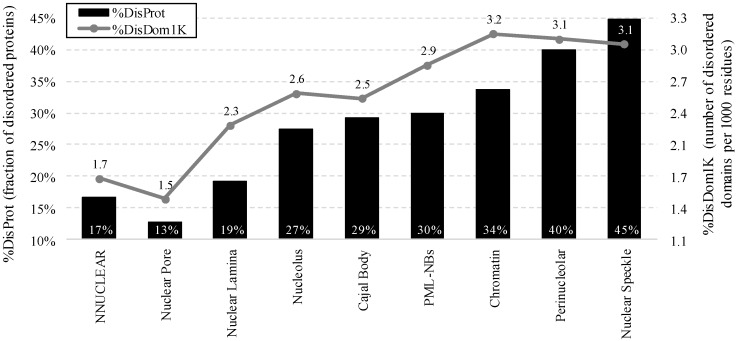
Fraction of disordered proteins (black bars) and normalized number of disordered domains (gray line) for the non-nuclear proteins from the NNUCLEAR dataset and proteins in the considered intra-nuclear compartment from the NUCLEAR*_ap_* dataset. Disorder was annotated with the consensus of Espritz and IUPred.

Figure 2 compares other characteristics of the intrinsic disorder including fraction of disordered proteins and normalized (by size of protein chains) fraction of disordered domains (corresponding Figure S3 is based only on the experimental annotations of localization).

We observe that most of the intra-nuclear compartments are enriched in disordered proteins; 27% of proteins in the nucleolus are disordered, around 30% in Cajal body, PML nuclear bodies and chromatin, and 40% or more in the perinucleolar compartment and nuclear speckle, compared to 17% in the non-nuclear proteins. Similarly, between 2.3 and 3.2 disordered domains per unit of 1000 residues can be found in the proteins from across all compartments, except for the nuclear pore (Figure 2). This is a large increase over the 1.7 domain per 1000 residues for the non-nuclear proteins.

**Figure 3:**
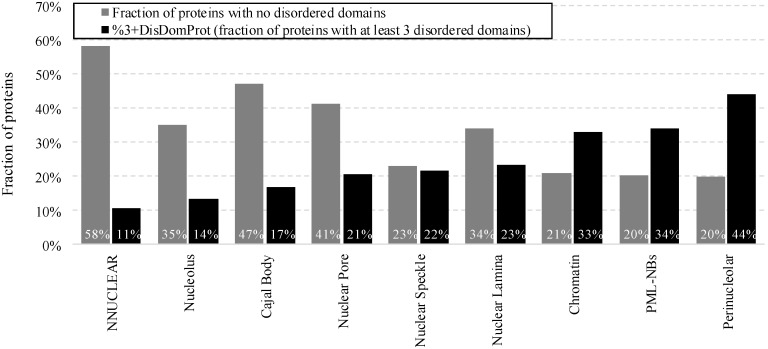
Fraction of proteins with no disordered domains (gray bars) and with at least 3 disordered domains (%3+DisDomProt) (black bars) for the non-nuclear proteins from the NNUCLEAR dataset and proteins in the considered intra-nuclear compartment from the NUCLEAR*_ap_* dataset. Disorder was annotated with the consensus of Espritz and IUPred.

Furthermore, Table S1 (see %DisDomProt values) reveals that between 59% (nuclear pore) and 80% (PML nuclear bodies and perinucleolar compartment) of proteins in the intra-nuclear compartments include disordered domains. Figure 3 compares the proportions of proteins that have no disordered domains and proteins that have at least three disordered domains (%3+DisDomProt) in each compartment and for the non-nuclear proteins (corresponding Figure S4 shows results on the NUCLEAR*_a_* dataset).

The majority of the non-nuclear proteins are structured and have no disordered domains and only 11% of these proteins have at least three such domains. In contrast, only between 20% (PML nuclear bodies and perinucleolar compartment) and 47% (Cajal body) of proteins localized in the intra-nuclear compartments have no disordered domains. Moreover, in several compartments (nuclear pore, speckle, and lamina) over 20% of proteins have three or more disordered domains, while in chromatin, PML nuclear bodies and perinucleolar compartment, these fractions reach well over 30%.

**Figure 4:**
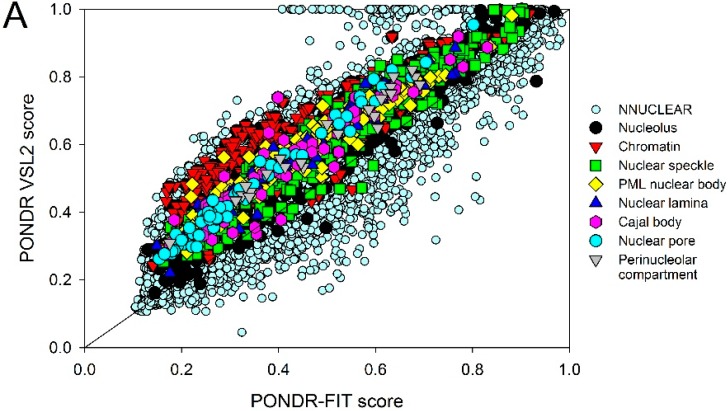

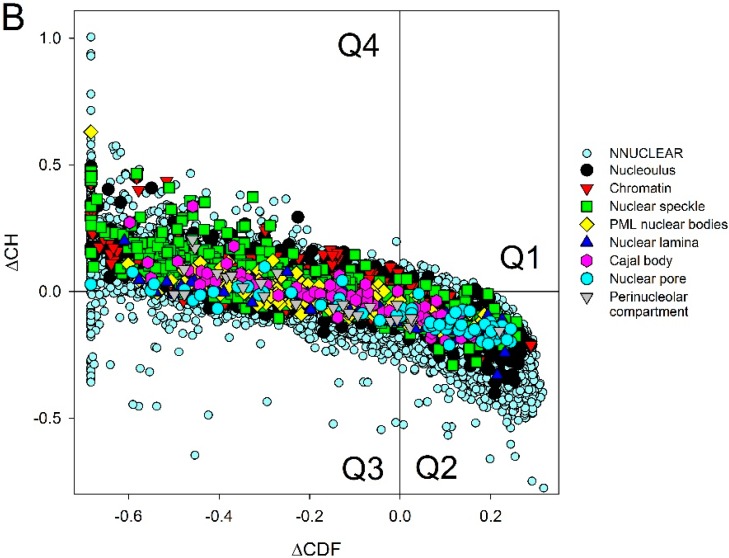
Abundance of intrinsic disorder in mouse proteins. (**A**) Per-proteins propensities for disorder (average of the corresponding per-residue propensities) evaluated by PONDR-FIT (*x*-axis) and by PONDR^®^ VSL2 (85) (*y*-axis) for the non-nuclear proteins from the NNUCLEAR dataset and proteins in the considered intra-nuclear compartment from the NUCLEAR*_ap_* dataset; (**B**) Evaluation of intrinsic disorder in mouse proteins (NNUCLEAR and NUCLEAR*_ap_* datasets) by CH-CDF analysis. In this figure, each point’s coordinates were computed as follows: the *y*-axis represents the distance of the protein in the CH-plot from the boundary, while the *x*-axis shows the average distance between the CDF boundary and the respective CDF curve. The figure is split into four quadrants/sections, with each quadrant representing a different family of predictions. Q1 shows proteins predicted to be ordered by CDFs but disordered by CH-plots. Q2 displays proteins that are completely ordered. Q3 shows proteins that were predicted as compact by CH-plots but disordered by CDFs (*i.e.*, hybrid proteins or putative molten globules). Finally, Q4 portrays proteins which both methods predicted to be disordered. Proteins found in different sub-nuclear compartments are indicated by differently colored symbols. In both plots, mouse non-nuclear proteins are shown by small light blue circles. Proteins in different nuclear compartments are indicated as follows: nucleolus —black circles; chromatin—red inverted triangles; nuclear speckles—green squares; PML nuclear bodies—yellow diamonds; nuclear lamina—blue triangles; Cajal bodies—pink hexagons; nuclear pore—cyan circles; and perinucleolar compartment—gray inverted triangles.

The point that mouse nuclear proteins analyzed in this study are mostly disordered is further illustrated by Figure 4A that compares the per-protein propensities for disorder (computed as average of the corresponding per-residue propensities) evaluated for the considered nuclear proteins by two additional predictors: PONDR-FIT [68] and by PONDR^®^ VSL2 [69]. Figure 4A indicates that the disorder prediction results obtained by both computational tools mostly agree with each other. Figure 4A and Table 2 show that mouse proteins in various nuclear compartments (except for the nuclear pore) are noticeably more disordered than the non-nuclear proteins. In fact, our analysis with PONDR-FIT (PONDR^®^ VSL2) indicates that the non-nuclear proteins are characterized by the mean per-protein propensity for disorder of 0.36 ± 0.17 (0.46 ± 0.18), whereas the corresponding values for the nuclear proteins range between 0.39 ± 0.17 (0.52 ± 0.17) for the nuclear lamina and 0.49 ± 0.15 (0.61 ± 0.15) for the perinucleolar compartment, excluding nuclear pore where they equal 0.34 ± 0.16 (0.47 ± 0.18) (Table 2).

**Table 2:** Evaluation of the disorder status for the non-nuclear proteins from the NNUCLEAR dataset and proteins in the considered intra-nuclear compartment from the NUCLEAR*_ap_* dataset.

Intra-Nuclear Compartments	Disorder Characteristics by the Per-Residue Predictors			Distribution within the CH-CDF Plot			
	PONDR-FIT ^a^	PONDR VSL2 ^a^	Two HT Consensus ^b^	Q1 (%)	Q2 (%)	Q3 (%)	Q4 (%)
Perinucleolar	0.49 ± 0.15	0.61 ± 0.15	36 ± 21	0.0	16.0	48.0	36.0
Nuclear speckle	0.48 ± 0.17	0.60 ± 0.17	38 ± 25	0.8	25.3	28.5	45.4
Chromatin	0.45 ± 0.15	0.61 ± 0.14	33 ± 21	0.7	15.7	33.9	49.7
PML	0.43 ± 0.14	0.57 ± 0.13	31 ± 19	0.0	22.4	48.7	28.9
Nucleolus	0.43 ± 0.16	0.55 ± 0.16	29 ± 22	1.0	34.6	29.4	35.0
Cajal body	0.42 ± 0.14	0.54 ± 0.13	30 ± 23	0.0	37.5	26.4	36.1
Nuclear lamina	0.39 ± 0.17	0.52 ± 0.17	22 ± 17	0.0	44.7	35.1	20.2
Nuclear pore	0.34 ± 0.16	0.47 ± 0.18	17 ± 16	0.0	61.9	23.8	14.3
Non-nuclear	0.36 ± 0.17	0.46 ± 0.18	20 ± 22	0.3	58.0	23.5	19.2
Total	0.37 ± 0.17	0.47 ± 0.18	21 ± 22	0.4	55.4	24.1	20.1

^a^ For PONDR-FIT and PONDR^®^ VSL2, Table 2 reports the average of the per-protein propensities for disorder and the corresponding standard deviations calculated for each dataset; ^b^ Content of predicted disordered residues in a given set of proteins based on a majority vote consensus of two high-throughput predictors, Espritz [70] and IUPred [71].

Therefore, according to these average per-protein propensities evaluated by PONDR-FIT, the intra-nuclear compartments can be arranged in the following order: perinucleolar compartment > nuclear speckles > chromatin > nucleolus = PML nuclear bodies > Cajal bodies > nuclear lamina > non-nuclear proteins > nuclear pore. These results are analogous to the results generated with the consensus of Espritz and IUPred (Table 2 and Figure 1).

The point that mouse nuclear proteins analyzed in this study are mostly disordered is further illustrated by Figure 4A that compares the per-protein propensities for disorder (computed as average of the corresponding per-residue propensities) evaluated for the considered nuclear proteins by two additional predictors: PONDR-FIT [68] and by PONDR^®^ VSL2 [69]. Figure 4A shows that the results of the disorder predictions obtained by these two computational tools mostly agree with each other. Figure 4A and Table 2 show that mouse proteins in various nuclear compartments (except for the nuclear pore) are noticeably more disordered than the non-nuclear proteins. In fact, our analysis with PONDR-FIT (PONDR^®^ VSL2) indicates that the non-nuclear proteins are characterized by the mean per-protein propensity for disorder of 0.36 ± 0.17 (0.46 ± 0.18), whereas the corresponding values for the nuclear proteins range between 0.39 ± 0.17 (0.52 ± 0.17) for the nuclear lamina and 0.49 ± 0.15 (0.61 ± 0.15) for the perinucleolar compartment, excluding nuclear pore where they equal 0.34 ± 0.16 (0.47 ± 0.18) (Table 2). Therefore, according to these average per-protein propensities evaluated by PONDR-FIT, the intra-nuclear compartments can be arranged in the following order: perinucleolar compartment > nuclear speckles > chromatin > nucleolus = PML nuclear bodies > Cajal bodies > nuclear lamina > non-nuclear proteins > nuclear pore. These results are analogous to the results generated with the consensus of Espritz and IUPred (Table 2 and Figure 1).

To understand how mouse nuclear proteins can be grouped into different disorder categories, we examine the details behind their distribution within the CH-CDF phase space. Figure 4B shows that, based on their location in this space, mouse nuclear proteins can be subdivided in three major groups, with the vast majority of proteins in almost all nuclear suborganelles (except for nuclear pore proteins) are predicted as disordered by both CDF and CH, or predicted as compact by CH but disordered by CDF (see also Table 2). In fact, these mostly disordered proteins are located within the quadrants 3 and 4 of the CH-CDF plot, respectively, and the overall content of such Q3 + Q4 proteins in different sub-nuclear compartments ranges from 84% in the perinucleolar compartment to 83.6% in chromatin, to 77.6% in PML nuclear bodies, to 73.9% in nuclear speckles, to 64.4% in nucleolus, to 62.5% in Cajal bodies, to 55.3% in nuclear lamina, and to 38.1% in nuclear pore. Therefore, the vast majority of nuclear proteins are expected to be either extended IDPs (behave as native pre-molten globules or native coils), or the potential native molten globules/hybrid proteins. Furthermore, statistical analysis (Pearson’s Chi-squared test with the Yates’ continuity correction) of these data revealed that the portion of Q4 proteins (*i.e.*, proteins that are expected to behave as native pre-molten globules or native coils) in Cajal body, chromatin, nucleolus, PML nuclear bodies, nuclear speckles, and perinucleolar compartment is significantly larger than the portions of proteins in other quadrants. Table 2 also shows that on average only ~29% of mouse proteins in various sub-nuclear domains are predicted as ordered by both CH and CDF and therefore are expected to be mostly ordered. There are only a very few proteins in this set which are predicted to be ordered by CDF and disordered by CH-plot analysis (typically, less than 1%). Figure 4B and Table 2 also show that the situation is very different for the mouse non-nuclear proteins, which are split between the four quadrants of the CH-CDF plot in the 0.3% (Q1):58% (Q2):23.5% (Q3):19.2% (Q4) proportion.

Altogether, our results that employ multiple methodologies clearly demonstrate that the intra-nuclear compartments (with the exception of the nuclear pore) are highly enriched in disordered residues, disordered domains, and disordered proteins.

### 2.3. Intrinsic Disorder in Proteins Co-Localized in Multiple Intra-Nuclear Compartments

**Figure 5:**
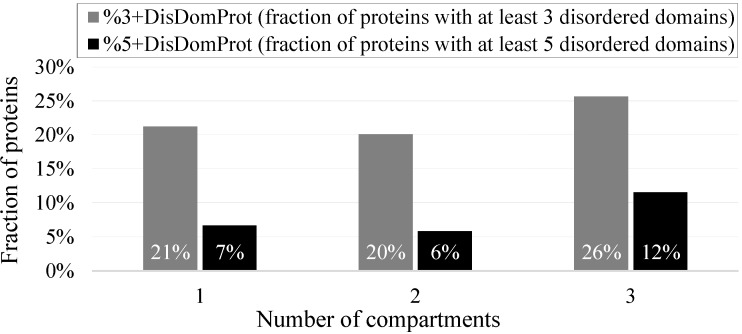
Fraction of proteins with 3 or more (%3+DisDomProt) and 5 or more (%5+DisDomProt) disordered domains for the nuclear proteins from the NUCLEAR*_ap_* dataset localized in 1, 2, and 3 intra-nuclear compartments. Disorder was annotated with the consensus of Espritz and IUPred.

Table S3 shows that while the majority of the nuclear proteins are assigned to a single intra-nuclear compartment, a large number is co-localized in multiple compartments. Approximately 19% of the nuclear proteins are assigned to two compartments and another 2.5% (78 proteins) to three compartments; only 10 proteins are associated with over three compartments and we have not considered them in our analysis due to the low count. The disorder content and fraction of disordered proteins is similar between the proteins assigned to one and to multiple compartments (Table S3). However, the fraction of proteins that have several disordered domains that are co-localized in three compartments is much higher compared to the proteins localized in one and two compartments (Figure 5). More specifically, about 26% proteins that are co-localized in three compartments have at least three disordered domains compared to 20% for proteins assigned to fewer compartments, which corresponds to the enrichment by 30%. The difference is even larger when considering proteins that have at least five disordered domains where the enrichment reaches 100%, from 6% to 12%. This result suggests the disordered domains play a role in the nuclear protein moonlighting.

Table S4 provides more detailed characterization of several mouse nuclear proteins that are co-localized in at least three sub-nuclear compartments and have at least five disordered domains. For this sample set, we predicted potential disorder-based binding sites using the ANCHOR algorithm [72,73] and also analyzed disorder content by three consensus approaches, MobiDB [74,75], PONDR-FIT [68], and the majority vote consensus of two high-throughput predictors, Espritz [70] and IUPred [71].

Table S4 shows a strong consensus between these three consensus-based approaches, which all show that proteins in the sample set are highly disordered and contain numerous potential disorder-based protein binding sites, AIBSs (ANCHOR-identified binding sites). In fact, the number of AIBSs ranges from 11 in transcription factor Sp7 (UniProt ID: Q8VI67) to 77 in Msx2-interacting protein (UniProt ID: Q62504) and the percentage of residues predicted to be involved in disorder-based protein interactions ranges from 12.7% in E3 SUMO-protein ligase RanBP2 (UniProt ID: Q9ERU9) to 51.1% in Msx2-interacting protein (UniProt ID: Q62504). On average, these selected nuclear multifunctional proteins are predicted to contain 16.4 AIBSs per 1000 residues, with 29.9% of residues in these proteins being predicted to be interaction-prone. The presence of so many AIBSs in all proteins analyzed here suggests that these mouse nuclear proteins that are co-localized in at least three sub-nuclear compartments and have at least five disordered domains are promiscuous binders that can be involved in various interactions with multiple unrelated partners and/or participate in the multivalent binding by wrapping around partners.

**Figure 6:**
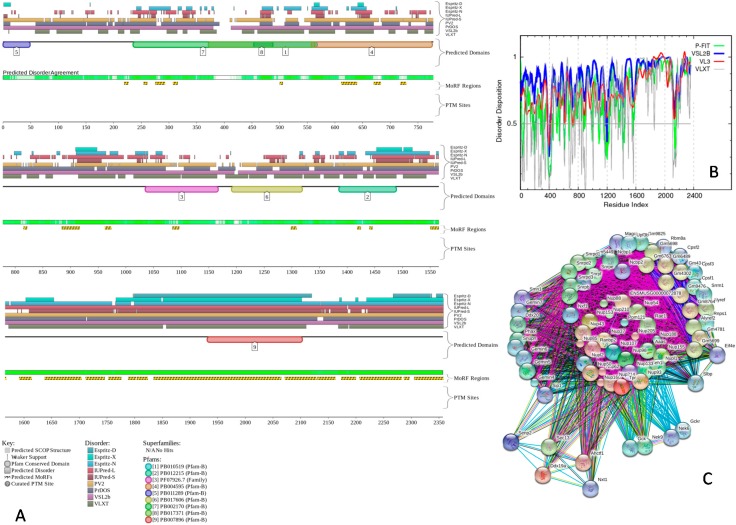
Abundance and functionality of intrinsic disorder in the nuclear pore complex-associated intra-nuclear coiled-coil protein TPR (UniProt ID: Q7M739). (**A**) Evaluation of the functional intrinsic disorder propensity by the D^2^P^2^ database (http://d2p2.pro/) [55]. In the corresponding plot, the colored bars at the top are representative of the location of disordered regions obtained from different disorder predictors (including IUPred-L, IUPred-S, Espritz-D, Espritz-N, Espritz-X, PONDR^®^ VSL2b, PONDR^®^ VLXT, PV2, and PrDOS). Location of predicted and known domains are shown by numbered colored bars. The white-and-green bars display the agreement in the predicted disorder among the aforementioned predictors, with green parts indicating regions that are disordered by consensus. The yellow bar indicates the location of the predicted disorder-based binding site (MoRF region), whereas red and yellow circles at the bottom of the plots show locations of phosphorylation and acetylation sites, respectively. Vertical dashed lines show actual positions of the phosphorylation sites; (**B**) Evaluation of the per-residue disorder propensity based on predictors from the PONDR family. A thin line at a score of 0.5 represents the disorder threshold between disorder (>0.5) and order (<0.5); (**C**) Analysis of the interactivity of the nuclear pore complex-associated intra-nuclear coiled-coil protein TPR (UniProt ID: Q7M739) by STRING computational platform [78]. STRING produces the network of predicted associations for a particular protein and its interactome. The nodes in this network are proteins, while the predicted or known functional associations are the edges. When predicting the associations, seven types of evidence are used, which are indicated in the resulting network by the differently colored lines. Here, a green line represents neighborhood evidence; a red line—the presence of fusion evidence; a purple line—experimental evidence; a blue line—co-occurrence evidence; a light blue line—database evidence; a yellow line—text mining evidence; a black line—co-expression evidence [78].

**Figure 7:**
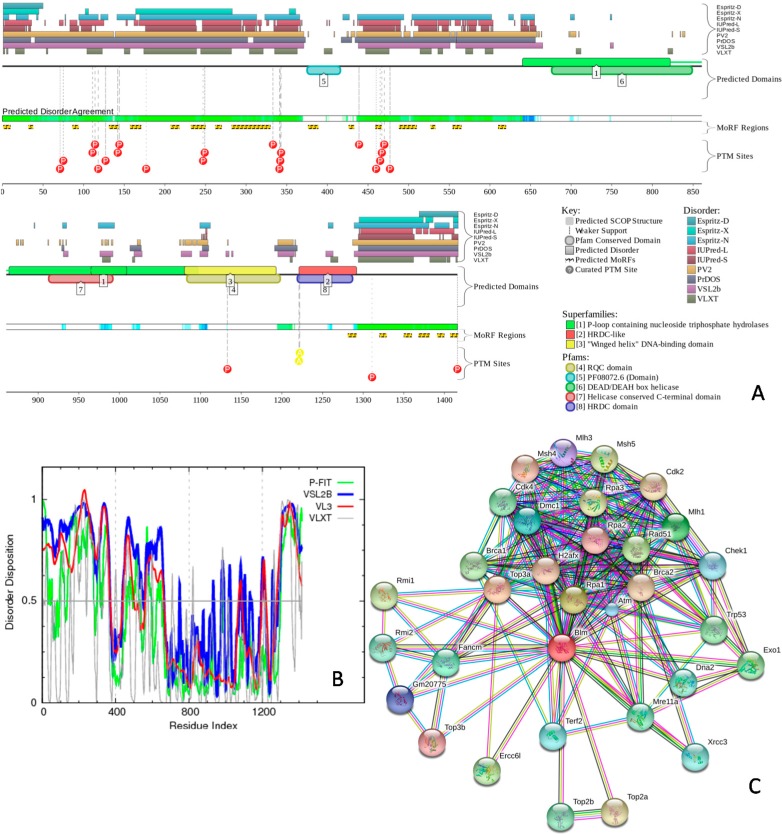
Functionality and prevalence of intrinsic disorder in the bloom syndrome protein homolog (UniProt ID: O88700). (**A**) Evaluation of the functional intrinsic disorder propensity by the D^2^P^2^ database (http://d2p2.pro/) [55]. In the corresponding plot, the colored bars at the top are representative of the location of disordered regions obtained from different disorder predictors (including IUPred-L, IUPred-S, Espritz-D, Espritz-N, Espritz-X, PONDR^®^ VSL2b, PONDR^®^ VLXT, PV2, and PrDOS). Location of predicted and known domains are shown by numbered colored bars. The white-and-green portion displays the agreement in the predicted disorder among the aforementioned predictors, with green parts indicating regions that are disordered by consensus. The yellow bar indicates the location of the predicted disorder-based binding site (MoRF region), whereas red circles with “P” inside and yellow circles with “A” inside at the bottom of the plots show locations of phosphorylation and acetylation sites, respectively. Vertical dashed lines show actual positions of these phosphorylation and acetylation sites; (**B**) Evaluation of the per-residue disorder propensity based on predictors from the PONDR family. A thin line at a score of 0.5 represents the disorder threshold between disorder (>0.5) and order (<0.5); (**C**) Analysis of the interactivity of the nuclear pore complex-associated intra-nuclear coiled-coil protein TPR (UniProt ID: Q7M739) by STRING computational platform [78]. STRING produces the network of predicted associations for a particular protein and its interactome. The nodes in this network are proteins, while the predicted or known functional associations are the edges. When predicting the associations, seven types of evidence are used, which are indicated in the resulting network by the differently colored lines. Here a green line represents neighborhood evidence; a red line—the presence of fusion evidence; a purple line—experimental evidence; a blue line—co-occurrence evidence; a light blue line—database evidence; a yellow line—text mining evidence; a black line—co-expression evidence [78].

To give a further illustration of the abundance and functionality of intrinsic disorder in nuclear proteins, Figure 6 and Figure 7 represent the results of the analysis of two of the proteins found in multiple sub-nuclear compartments, nuclear pore complex-associated intra-nuclear coiled-coil protein TPR (UniProt ID: Q7M739), and bloom syndrome protein homolog (UniProt ID: O88700) by D^2^P^2^, multiple per-residue predictors of the PONDR family, and STRING. These two proteins were selected from the subset of nuclear proteins shown in Table 3 as one of the most disordered and one of the least disordered proteins, respectively. The results of the analogous analyses for the remaining proteins from Table S4 are presented in Figures S5–S11.

First, the data corresponding to these two proteins were retrieved from the D^2^P^2^ database [55] that contains information on predicted disorder and selected disorder-related functions. Besides providing information on the distribution of disordered regions predicted by nine tools in a query protein, the D^2^P^2^ visual console is further enhanced by providing information on the location of predicted disorder-based protein binding sites and on the curated sites of various posttranslational modifications (see Materials and Methods). This analysis confirmed that the selected nuclear proteins are predicted to possess long disordered regions, contain numerous binding sites and multiple sites of various posttranslational modifications (see Figure 6A and Figure 7A, and Figures S5–S11). The fact that disordered domains/regions of these nuclear proteins have several posttranslational modification sites is in agreement with the well-known fact that phosphorylation [76] and many other enzymatically catalyzed posttranslational modifications are preferentially located within the IDPRs [77].

**Table 3:** Number of proteins and hubs in the intra-nuclear compartments.

Intra-Nuclear Compartment	Number of Proteins in NUCLEAR*_a_*	Number of Proteins in NUCLEAR*_ap_*	PPI_NUCLEAR*ap*_		
			Number of Proteins	Number of Hubs	Number of Intra-Compartment Hubs
Cajal Body	49	72	50	15	11
Chromatin	323	828	500	192	145
Nuclear Lamina	77	94	70	19	14
Nuclear Pore	51	63	49	15	11
Nuclear Speckle	403	632	459	138	104
Nucleolus	598	1860	1227	392	335
Perinucleolar	24	25	24	14	9
PML nuclear bodies	91	187	140	71	49

Next, the per-residue intrinsic disorder propensities of the selected two proteins (see Figure 6B and Figure 7B) and remaining proteins from Table S4 (see Figures S5–S11) were analyzed by various predictors from the PONDR family, such as PONDR^®^ FIT [68], PONDR^®^ VSL2 [69], PONDR^®^ VLXT [79], and PONDR^®^ VL3 [80]. Here, a disorder score above 0.5 corresponds to disordered residues/regions. This analysis provides additional support to the idea that the selected mouse nuclear proteins co-localized in several nuclear compartments are predicted to contain numerous IDPRs.

Finally, the interactivity of these selected nuclear proteins was further evaluated by STRING (Search Tool for the Retrieval of Interacting Genes) database [78]. This database produces the network of predicted associations for a particular protein and its interactome (see Materials and Methods). Figure 6C and Figure 7C represent the results of STRING analysis of nuclear pore complex-associated intra-nuclear coiled-coil protein TPR and bloom syndrome protein homolog, whereas Figures S5–S11 for the remaining proteins from Table S4. This analysis shows that all proteins are expected to have rather well developed interactomes, likely serving as hub proteins in their functional protein-protein interaction networks (PPI). It was pointed out that intrinsic disorder represents an important feature defining the ability of a hub protein to be a promiscuous binder [43,59,81,82,83,84,85]. In fact, intrinsic disorder is always associated with the ability to serve as a hub, and many hubs are intrinsically disordered or contain functional IDPRs, whereas partners of ordered hubs are known to be abundantly disordered [43,59,81,82,83,84,85].

### 2.4. Protein-Protein Interactions and Intrinsic Disorder in Intra-Nuclear Compartments

Motivated by the abundance of protein-binding regions and the large sizes of interactomes of proteins that are co-localized in multiple intra-nuclear compartments (Figure 6 and Figure 7, and Figures S5–S11), we analyze PPIs in the considered compartments.

**Figure 8:**
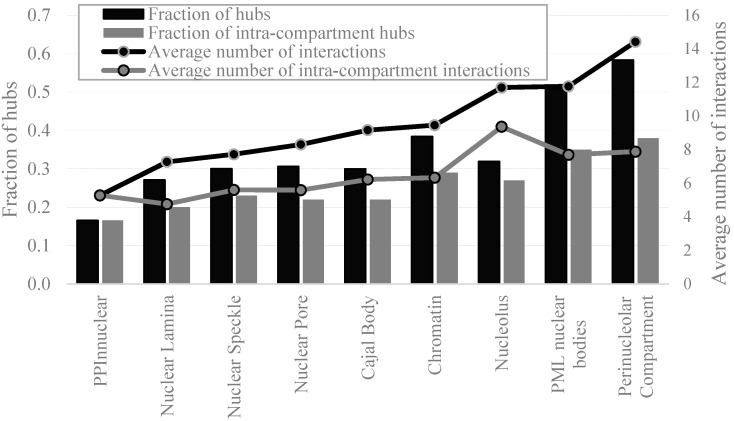
Fraction of hubs (black bars), intra-compartment hubs (gray bars), average number of interactions (black line), and average number of intra-compartments interactions (gray line) for the non-nuclear proteins from the PPI_NNUCLEAR_ dataset and proteins in the considered intra-nuclear compartment from the PPI_NUCLEAR*ap*_ dataset. Disorder was annotated with the consensus of Espritz and IUPred.

Table 3 shows number of proteins and hubs in the intra-nuclear compartments, whereas Table S2 summarizes results for the nuclear and non-nuclear proteins that are included in the PPI network (PPI_NUCLEAR*ap*_ and PPI_NNUCLEAR_ datasets). We also count intra-compartment hubs, *i.e.*, proteins that are hubs when excluding their interactions with proteins located in other intra-nuclear compartments (interactions across compartments). The nuclear proteins are characterized by a substantial enrichment in hubs and average (per protein) number of PPIs. While the non-nuclear proteins include about 17% of hubs, proteins localized in the intra-nuclear compartments have between 27% (nuclear lamina) and 58% of hubs (perinucleolar compartment) (Figure 8). The average number of PPIs for the non-nuclear proteins is at 5.3 and it goes up to between 7.3 and 14.4 for the proteins localized in the intra-nuclear compartments (Figure 8).

Even when excluding the interactions across compartments, the fraction of intra-compartment hubs is substantially higher in all nuclear compartments, between 20% and 38%, as compared to the fraction of hubs among the non-nuclear proteins (Figure 8). Similarly, the average number of PPIs for all compartments except for the nuclear lamina is higher even when excluding the inter-compartment interactions. The corresponding highest enrichment in the PPIs is for the nucleolus, PML nuclear bodies, and perinucleolar compartment (Figure 8).

**Figure 9:**
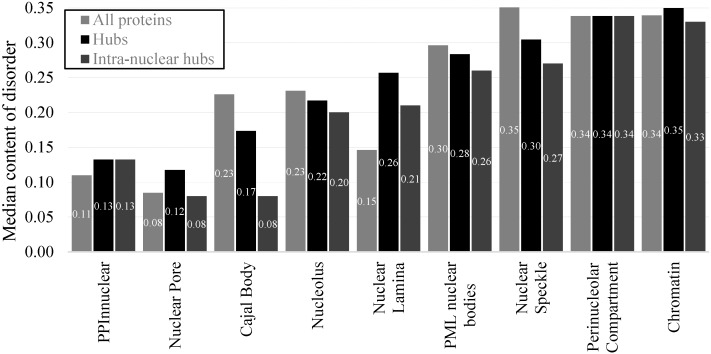
Median disorder content for all proteins (light gray bars), hubs (black bars), and intra-nuclear hubs (dark gray bars) from a given protein set including the non-nuclear proteins in the PPI_NNUCLEAR_ dataset and proteins in the considered intra-nuclear compartment from the PPI_NUCLEAR*ap*_ dataset. Disorder was annotated with the consensus of Espritz and IUPred.

Table S5 provides details of the abundance of the disorder in the hub proteins across all considered intra-nuclear compartments and the non-nuclear proteins. Interestingly, the non-nuclear hubs are represented by heightened amounts of disorder when viewed against the overall population of non-nuclear proteins (median disorder content of 0.13 *vs.* 0.11, Figure 9), which agrees with previously reported data [83]. We observe that the proteins localized in the intra-nuclear compartments have already high amounts of disordered and similarly high disorder content is characteristic for the corresponding hubs (Figure 9). Hubs in all compartments, except for the nuclear pore, are substantially enriched in the disorder compared to both non-nuclear hubs and all non-nuclear proteins. Their median disorder content varies between 0.17 (Cajal body) and 0.35 (chromatin). Over 75% of hubs have disordered domains and over 30% of hubs are disordered in several intra-nuclear compartments including nuclear speckle, PML nuclear bodies, perinucleolar compartment and chromatin (Table S5). Figure 9 also shows that the intra-nuclear hubs (when we exclude the inter-nuclear compartment interactions) are also enriched in disorder for the majority of the compartments, except for the nuclear pore and cajal body. The intra-nuclear hubs in these two compartments are depleted in disorder as compared to all hubs, which means that disorder is likely involved in the inter-compartment interactions. The disorder content in the other six inter-nuclear compartments is comparable between all hubs and the intra-nuclear hubs.

We conclude that the density of the PPIs for the nuclear proteins is unusually high. The intra-nuclear compartments are rich in hub proteins and these hubs are substantially enriched in disorder and include a large fraction of disordered proteins when compared to the non-nuclear hubs. When excluding interactions across nuclear compartments, we still observe enrichment in the abundance of hub proteins and their disorder content for the majority of the compartments.

## 3. Materials and Methods

### 3.1. Datasets

Our analysis compares nuclear proteins and subgroups of nuclear proteins localized in specific intra-nuclear compartments with other proteins in the same proteome. Thus, we collected a complete set of nuclear and non-nuclear proteins. We use mouse proteome due to the availability of well-annotated intra-nuclear compartments for its nuclear proteins and a relatively comprehensive PPI network. We collected nuclear proteins from the intra-nuclear compartment protein database Nsort/DB [86]. This comprehensive database aggregates data from multiple sources including Nuclear Protein Database (NPD) [87], Nuclear Proteome Database (NOPdb) [88], Nuclear Matrix Protein Database (NMPdb) [89], UniProt [90] and Human Protein Reference Database (HPRD) [91]. The complete nuclear proteome contains 3566 proteins where 1285 proteins are assigned into at least one intra-nuclear compartment based on experimental evidence [86] (they compose NUCLEAR*_a_* dataset, see Table 1) and another 1720 proteins for which the assignment was predicted [92]. The NUCLEAR*_ap_* dataset combines proteins with annotated and predicted intra-nuclear localization. We mapped the complete set of nuclear proteins into the complete mouse proteome to create the set of non-nuclear proteins. The mouse proteome was collected from UniProt (release from August 2013) and we excluded protein fragments. The mapping was done primarily based on the accession numbers; in 233 cases where accession numbers did not match we used sequence identity. The resulting NNUCLEAR dataset includes 29,863 mouse proteins that exclude the nuclear proteins.

Furthermore, we analyzed differences in PPIs between nuclear proteins, including subsets of nuclear proteins localized in specific intra-nuclear compartments, and the non-nuclear proteins. We collected the mouse PPI network from the Mentha database (release from October 2013) [93] which includes 8206 proteins that have at least one interaction. We mapped these proteins into the NNUCLEAR and NUCLEAR*_ap_* datasets and collected a set of 5687 non-nuclear proteins (PPI_NNUCLEAR_ dataset) and 2519 nuclear proteins (PPI_NUCLEAR*ap*_ dataset) that are included in the PPI network, respectively (Table 1). We annotate hubs [83], highly connected proteins in the PPI networks, in the PPI_NNUCLEAR_ and PPI_NUCLEAR*ap*_ datasets, including the intra-nuclear compartments in the latter dataset. Based on previously reported criteria [94,95,96], hubs are defined as the top 20% most connected proteins in the PPI network based on the PPI dataset. The number of hubs in the PPI_NNUCLEAR_ dataset is 942. The number of proteins and hubs in individual intra-nuclear compartments is summarized in Table 2; note that we cannot annotate hubs in the NUCLEAR*_a_* and NUCLEAR*_ap_* datasets.

### 3.2. Annotation and Characterization of Intrinsic Disorder

We generated annotations of intrinsic disorder using a majority vote consensus of two high-throughput predictors. Use of the consensus was shown to lead to an increase in the predictive performance compared to the use of a single predictor [97,98,99]. We used three versions of Espritz [70], which were designed to predict disorder in proteins annotated based on X-ray crystal structures, NMR-derived structures, and the Disprot database [100]; and two versions of IUPred [71], for prediction of long and short disordered regions. A recent large-scale assessment shows that these predictors offer good predictive performance, with AUC values around 0.77 [97]. We also note that the same consensus was recently utilized in a few related works [56,101,102]. We computed the putative annotations of the disorder for each residue and aggregated them for a given protein or a group of proteins using the following measures:

- Disorder content: fraction of disordered residues in a given protein
- %DisProt (fraction of disordered proteins): fraction of proteins with disorder content ≥0.4 in a given protein set
- %DisDomProt (fraction of proteins with disordered domains): fraction of proteins that have at least one long disorder region (≥30 residues long) in a given protein group. Such long regions are recognized as functional protein domains [52,103,104], which means that proteins with the long disorder regions are likely to carry functions through disorder
- %3+DisDomProt (fraction of proteins with at least three disordered domains)
- %5+DisDomProt (fraction of proteins with at least five disordered domains)
- %DisDom1 K (number of disordered domains per 1000 residues).This measure normalizes the count of long disordered regions to a size of a given protein or a set of proteins to accommodate for a potential bias that longer proteins may have more disordered domains because of their length.

We calculated these measures for the non-nuclear proteins in NNUCLEAR and PPI_NNUCLEAR_, and the nuclear proteins in each intra-nuclear compartment in NUCLEAR*_a_*, NUCLEAR*_ap_*, and PPI_NUCLEAR*ap*_. We also did the same calculation for hubs from PPI_NNUCLEAR_ and each compartment in PPI_NUCLEAR*ap*_.

To further boost confidence of the putative annotations of disorder, we also calculated disorder content for the mouse non-nuclear proteins in the NNUCLEAR dataset and the nuclear proteins in each intra-nuclear compartment in NUCLEAR*_ap_* dataset using a different metapredictor PONDR-FIT [68]. This method was shown to be moderately more accurate than each of its six component predictors, PONDR^®^ VSL2 [69], PONDR^®^ VLXT [79], FoldIndex [105], PONDR^®^ VL3 [80], TopIDP [106], and IUPred [71].

### 3.3. Disorder Sub-Classification of Mouse Proteins Based on the Charge/Hydropathy-Cumulative Distribution Function Analysis

The charge/hydropathy-cumulative distribution function (CH-CDF) analysis represents a simple graphic way of intrinsic disorder classification in whole proteins. In the resulting CD-CDF plot, each point corresponds to a protein with coordinates computed in the following manner: the *Y* coordinate represents the distance between the boundary and the corresponding protein in the CH-plot (charge-hydropathy plot) [45,53], while the *X* coordinate represents the average distance between the CDF boundary and the corresponding cumulative distribution function (CDF) curve [53,107,108,109]. The use of the CH-CDF plot utilizes the principle difference in the logistics of how the two component binary predictors evaluate the predisposition of a given protein to be disordered or ordered or overall. Here, the CH-plot serves as a linear classifier utilizing the charge and hydropathy of a particular protein sequence [45,53]. On the other hand, analysis of the CDF relies on the output of a PONDR^®^ predictor, which is a nonlinear disorder classifier trained to distinguish order and disorder based on a significantly larger sequence feature space [53]. According to these methodological differences, the CH-plot can discriminate proteins with compact conformations (well-structured globular and molten globule-like proteins) from proteins with substantial amount of extended disorder (pre-molten globules and random coils), whereas CDF analysis may discriminate all types of IDPs, including molten globules, from the ordered proteins. The aforementioned methodological differences constitute a means for the simple visual sub-classification of proteins into ordered proteins, IDPs with extended disorder, molten globular compact IDPs, and hybrid proteins containing ordered domains and IDPRs. In the resulting CH-CDF plot, positive *Y* values correspond to proteins predicted to be extended IDPs by the CH-plot analysis, while negative *Y* values indicate proteins predicted to be compact. Meanwhile, positive *X* values indicate proteins predicted to be ordered by CDF analysis, and negative *X* values represent predicted IDPs. The resultant quadrants of CDF-CH plots can therefore be summarized as follows: Q1 contains proteins predicted to be ordered by CDFs but disordered by CH-plots. Q2 displays proteins predicted to be completely ordered by both methods. Q3 shows proteins that were predicted as compact by CH-plots but disordered by CDFs (*i.e.*, hybrid proteins or putative molten globules). Finally, Q4 contains proteins which both methods predicted to be disordered [109,110].

### 3.4. Disorder and Disorder—Based Functional Analysis of Selected Mouse Nuclear Proteins with Consensus Disorder Predictors, PONDR Predictors, D^2^P^2^, STRING, and ANCHOR

Disorder evaluations for several selected proteins (which are mouse nuclear proteins that are co-localized in at least three sub-nuclear compartments and have at least five disordered domains) are further analyzed by three consensus-based computational tools for evaluation of intrinsic disorder, PONDR-FIT [68] and the majority vote consensus approach that were described above and MobiDB [74,75]. The MobiDB database (http://mobidb.bio.unipd.it/) [74,75], that generates consensus disorder scores by aggregating the output from ten predictors, such as two versions of IUPred [71], two versions of ESpritz [70], two versions of DisEMBL [111], JRONN [112], PONDR^®^ VSL2B [80,113], and GlobPlot [114]. Use of consensuses for evaluation of intrinsic disorder is motivated by empirical observations that this leads to an increase in the predictive performance compared to the use of a single predictor [97,98,99].

Per-residue disorder distribution was evaluated by PONDR predictors, including PONDR^®^ FIT [68], PONDR^®^ VSL2 [69], PONDR^®^ VLXT [79], and PONDR^®^ VL3 [80]. Here, a disorder score above 0.5 indicated disordered residues/regions. These four predictors were chosen for several reasons. PONDR^®^ VSL2 was chosen due to being one of the best standalone predictors [69,98,115]. PONDR^®^ VLXT is useful for identifying disorder-based interaction sites since it is sensitive to minute aberrations in local sequences [79]. PONDR^®^ VL3 was used since it is very accurate when evaluating long disordered regions [80], whereas PONDR-FIT is already described above. Again, we use multiple disorder predictors here to increase confidence of our results.

For the same selected mouse nuclear proteins co-localized in several sub-nuclear compartments, disorder evaluations together with important disorder-related functional information were retrieved from the D^2^P^2^ database (http://d2p2.pro/) [55]. D^2^P^2^ is a database of predicted disorder that serves as a collaborative resource for pre-computed disorder predictions. Its contents include results for a large library of proteins from completely sequenced genomes [55]. D^2^P^2^ database uses outputs of IUPred [71], PONDR^®^ VLXT [79], PrDOS [116], PONDR^®^ VSL2B [80,113], PV2 [55], and ESpritz [70]. The database is further supplemented by data concerning location of various curated posttranslational modifications and predicted disorder-based protein binding sites.

Additional functional information for these proteins was retrieved using the STRING platform (Search Tool for the Retrieval of Interacting Genes; http://string-db.org/) which offers both predicted and information on the interactions of a protein of interest [78]. For a query protein, STRING produces the network of predicted associations with a particular group of proteins. The nodes in this network are proteins, while the predicted or known functional associations are the edges. When predicting the associations, seven types of evidence are used, which are indicated in the resulting network by the differently colored lines. Here a green line represents neighborhood evidence; a red line—the presence of fusion evidence; a purple line—experimental evidence; a blue line—co-occurrence evidence; a light blue line—database evidence; a yellow line—text mining evidence; a black line—co-expression evidence [78]. In our analysis, the most stringent criteria were used for selection of interacting proteins by choosing the highest cut-off of 0.9 as the minimal required confidence level.

Potential protein binding sites in disordered regions of selected mouse nuclear proteins that are co-localized in at least three sub-nuclear compartments and have at least five disordered domains were identified by the ANCHOR algorithm [72,73]. This algorithm utilizes the pair-wise energy estimation approach originally used by IUPred [71,117], This approach acts on the hypothesis that long regions of disorder include localized potential binding sites which are not capable of folding on their own due to not being able to form enough favorable intrachain interactions, but can obtain the energy to stabilize via interaction with a globular protein partner [72,73]. The ANCHOR algorithm generates regions with a high likelihood to be binding sites, which are referred to as ANCHOR-indicated binding site (AIBS).

### 3.5. Statistical Analysis

We compared the median and mean disorder content and the values of %DisProt, %DisDomProt, %3+DisDomProt, %5+DisDomProt, and %DisDom1K computed for the nuclear proteins in the intra-nuclear compartments from NUCLEAR*_a_* or NUCLEAR*_ap_* (PPI_NUCLEAR*ap*_) with the corresponding values for the non-nuclear proteins from the NNUCLEAR (PPI_NNUCLEAR_) dataset. We assessed statistical significance of the differences in these values between proteins in a given intra-nuclear compartment and the corresponding non-nuclear proteins following the procedure from Refs [56,118]. We determined the significance between two sets of values of a given measurement computed over ten subsets of randomly chosen half of proteins in the corresponding two protein sets. More specifically, we selected half of the proteins in a given intra-nuclear compartment and the same number of non-nuclear proteins with similar size (with ±10% tolerance) compared to the selected nuclear proteins; we repeated that ten times. We matched the size since the amount of disorder was shown to be dependent on the protein chain length in eukaryotes [101]. We used *t*-test for measurements that follows normal distribution as evaluated with the Anderson-Darling test [119] at the 0.05 significance; otherwise, we used the Wilcoxon rank sum test [120]. We assumed that a given difference is significant if the *p*-value <0.01.

## 4. Conclusions

This work provides support to the idea of the functional importance of IDPs in the cell nucleus and shows that many sub-nuclear organelles in nuclei of mouse cells are enriched in IDPs and hybrid proteins containing disordered and ordered domains. We also show that the mouse nuclear proteins are very promiscuous binders possessing both large quantities of potential disorder-based interaction sites and the ability of a single such site to be involved in a large number of interactions. In fact, we reveal that intrinsic disorder (overall content, number of disordered proteins, and number of disordered domains) is enriched in the majority of the mouse intra-nuclear compartments, except for the nuclear pore and lamina. Intra-nuclear compartments are depleted in proteins that lack disordered domains and enriched in proteins that have multiple disordered domains. Proteins that are co-localized in multiple intra-nuclear compartments are more likely to have multiple disordered domains, which could be related to their moonlighting functions. Protein-protein interaction networks in the mouse intra-nuclear compartments are denser and include more hub proteins compared to the non-nuclear proteins. Hubs in the intra-nuclear compartments (except for the nuclear pore) are enriched in disorder and include more disordered proteins compared with non-nuclear hubs and non-nuclear proteins. These results were obtained based on analysis with several different tools that provided unanimous support for the above observations.

Since the dataset analyzed here contains more than 3000 entries, it is physically impossible to provide even a brief description of all these mouse nuclear proteins. However, our observations are in agreement with known experimental and computational data for several nuclear proteins. For example, the exceptional functional importance of intrinsic disorder has been shown for histones [20,121,122,123,124,125,126,127,128,129,130,131,132,133,134,135,136,137,138], nucleoporins [139,140,141,142], as well as for ribosomal [102] and spliceosomal proteins [143,144]. These proteins are found in chromatin, nuclear pore, nucleolus, and Cajal bodies, respectively. Intrinsic disorder is also known to play an important role in function of transcription factors [145,146].

Lastly, we comment on the potential role of intrinsic disorder in assembly and disassembly of the sub-nuclear membrane-less organelles. Recently, it has been proposed that IDPs may play an important role in driving the intracellular liquid-liquid phase separations generating various nucleoplasmic and cytoplasmic membrane-less organelles [147]. Although these organelles are found in different cellular and nuclear locations, and although they are composed of rather different proteins and nucleic acids, it is believed that such membrane-less organelles are formed via a common mechanism related to the intracellular phase transitions [42]. Such transitions occur due to the changes to the structure and solvent properties of water that are driven by macromolecules, and can usually be found in high concentrations of macromolecular solutes. This happens because the solution exists as a single phase at low macromolecule concentrations but experiences phase separation at higher concentrations [148]. An earlier study revealed that several of the proteins that play a role in the formation of the nucleoplasmic or cytoplasmic membrane-less organelles are in fact intrinsically disordered [66,147]. It was also hypothesized that because the IDPs are known to be engaged in various weak interactions of a different physico-chemical nature and because these proteins are commonly seen in different cytoplasmic and nuclear membrane-less organelles, IDPs might serve as perfect regulators and controllers of the formation of these organelles via the aforementioned phase separation [147]. Data reported in our work provide indirect support to this hypothesis by showing that many membrane-less sub-nuclear organelles are enriched in IDPs and by emphasizing that these disordered nuclear proteins are highly promiscuous binders.

## Supplementary Materials

Supplementary materials can be found at http://www.mdpi.com/1422-0067/17/1/24/s1.
