# Supplementary material for: Compartmentalization and Functionality of Nuclear Disorder: Intrinsic Disorder and Protein-Protein Interactions in Intra-Nuclear Compartments
Source: Int J Mol Sci. 2015 Dec 25;17(1):24. doi: 10.3390/ijms17010024 (PMC4730271; doi:10.3390/ijms17010024)
Supplement: Supplementary file 1 [file ijms-17-00024-s001.pdf]

Supplementary Materials: Compartmentalization and Functionality of Nuclear Disorder: Intrinsic Disorder and Protein-Protein Interactions in Intra-Nuclear Compartments

Fanchi Meng, Insung Na, Lukasz Kurgan and Vladimir N. Uversky

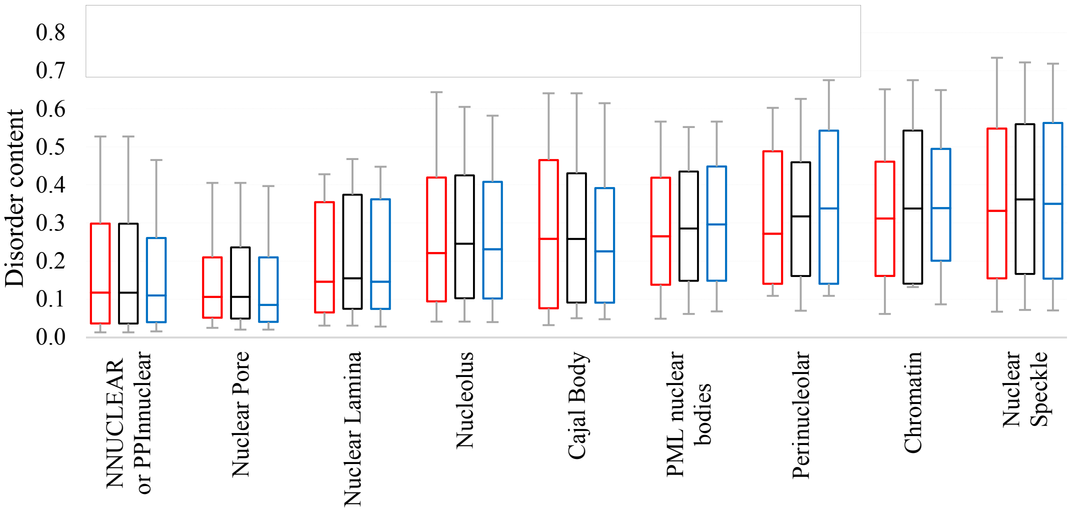

Figure S1. Disorder content for the nuclear proteins with annotated intra-nuclear compartments (red boxplots; NUCLEAR$_{a}$ dataset), with annotated and predicted intra-nuclear compartments (black boxplots; NUCLEAR$_{ap}$ dataset), and with annotated and predicted intra-nuclear compartments in the PPI network (blue boxplots; PPI$_{NUCLEARap}$ dataset). The leftmost set of three boxplots shows all non-nuclear proteins (red and black boxplots; NNUCLEAR dataset) and the non-nuclear proteins in the PPI network (blue boxplots; PPI$_{NNUCLEAR}$ dataset). The box plots include the first quartile, median andthird quartile while whiskers correspond to the 10th and 90th centiles of the disorder content of proteins in a given protein set. Disorder was annotated with the consensus of Espritz and IUPred.

Table S1. Disorder content, fraction of disordered proteins, and abundance of disordered domains for the nuclear proteins in specific intra-nuclear compartments from the NUCLEAR$_a$ and NUCLEAR$_{ap}$ datasets and for the non-nuclear proteins from the NNUCLEAR dataset. Disorder was annotated with the consensus of Espritz and IUPred.

		**Disorder Content**				**Fraction of Disordered Proteins %DisProt**		**Fraction of Proteins with Disordered Domains**						**Normalized Number of Disordered Domains %DisDom1K**	
**Localization**	**Dataset**	**Median**		**Mean**				**%DisDomProt**		**%3+DisDomProt**		**%5+DisDomProt**			
		**Value**	***p*-Value**	**Value**	***p*-Value**	**Value**	***p*-Value**	**Value**	***p*-Value**	**Value**	***p*-Value**	**Value**	***p*-Value**	**Value**	***p*-Value**
Non-nuclear	NNUCLEAR	0.12	-	0.20	-	0.17	-	0.42	-	0.11	-	0.04	-	1.72	-
Nuclear Pore	NUCLEAR$_a$	0.11	0.002	0.17	0.007	0.14	0.002	0.57	0.182	0.16	0.082	0.08	0.002	1.49	0.002
	NUCLEAR$_{ap}$	0.11	0.002	0.17	0.648	0.13	0.002	0.59	0.764	0.21	0.002	0.10	0.322	1.51	0.867
Nuclear Lamina	NUCLEAR$_a$	0.15	0.550	0.21	0.984	0.17	0.619	0.65	0.019	0.19	0.703	0.10	0.168	2.14	0.417
	NUCLEAR$_{ap}$	0.15	0.069	0.22	0.111	0.19	0.207	0.66	0.009	0.23	0.030	0.13	0.002	2.25	0.009
Nucleolus	NUCLEAR$_a$	0.22	0.000	0.29	0.000	0.26	0.000	0.65	0.000	0.16	0.000	0.04	0.751	2.37	0.002
	NUCLEAR$_{ap}$	0.25	0.000	0.29	0.000	0.27	0.000	0.65	0.000	0.14	0.000	0.03	0.321	2.58	0.000
Cajal Body	NUCLEAR$_a$	0.26	0.000	0.30	0.001	0.35	0.001	0.53	0.035	0.20	0.002	0.06	0.002	2.32	0.015
	NUCLEAR$_{ap}$	0.26	0.002	0.30	0.000	0.29	0.002	0.53	0.000	0.17	0.000	0.06	0.002	2.52	0.000
PML-NBs	NUCLEAR$_a$	0.27	0.000	0.29	0.000	0.30	0.002	0.76	0.000	0.31	0.000	0.09	0.210	2.68	0.000
	NUCLEAR$_{ap}$	0.29	0.002	0.31	0.000	0.30	0.000	0.80	0.000	0.34	0.000	0.12	0.002	2.86	0.000
Chromatin	NUCLEAR$_a$	0.31	0.000	0.34	0.000	0.31	0.000	0.80	0.000	0.33	0.000	0.14	0.000	2.98	0.002
	NUCLEAR$_{ap}$	0.32	0.000	0.33	0.000	0.34	0.002	0.79	0.000	0.33	0.000	0.14	0.000	3.17	0.000
Perinucleolar	NUCLEAR$_a$	0.27	0.002	0.35	0.000	0.38	0.000	0.79	0.002	0.42	0.002	0.04	0.002	3.21	0.000
	NUCLEAR$_{ap}$	0.34	0.001	0.36	0.000	0.40	0.002	0.80	0.002	0.44	0.002	0.08	0.002	3.12	0.000
Nuclear Speckle	NUCLEAR$_a$	0.33	0.000	0.38	0.000	0.44	0.000	0.77	0.000	0.23	0.000	0.07	0.002	2.88	0.000
	NUCLEAR$_{ap}$	0.36	0.002	0.38	0.000	0.45	0.000	0.77	0.000	0.22	0.000	0.06	0.003	3.07	0.000

Table S2. Fraction of hub and intra-compartment hub proteins, average number of all and intra-compartment protein-protein interactions (PPIs), disorder content, fraction of disordered proteins, and abundance of disordered domains for the nuclear proteins in specific intra-nuclear compartments from the PPI$_{\text{NUCLEAR}ap}$ datasets and for the non-nuclear proteins from the PPI$_{\text{NNUCLEAR}}$ dataset. Disorder was annotated with the consensus of Espritz and IUPred.

Localization	All PPIs		PPIs When Excluding Interactions across Compartments		Disorder Content		Fraction of Disordered Proteins %DisProt	Fraction of Proteins with Disordered Domains			Normalized Number of Disordered Domains %DisDom1K
	Fraction of Hubs	Average Number of PPIs	Fraction of Intra-Compartment Hubs	Average number of Intra-Compartment PPIs	Median	Mean		%DisDomProt	%3+DisDomProt	%5+DisDomProt	
Non-nuclear	0.17	5.28	0.17	5.28	0.11	0.18	0.14	0.46	0.12	0.04	1.62
Nuclear Pore	0.31	8.31	0.22	5.59	0.08	0.15	0.10	0.51	0.16	0.08	1.39
Nuclear Lamina	0.27	7.27	0.20	4.76	0.15	0.22	0.19	0.63	0.23	0.13	2.31
Nucleolus	0.32	11.69	0.27	9.36	0.23	0.28	0.26	0.66	0.14	0.03	2.51
Cajal Body	0.30	9.16	0.22	6.22	0.23	0.27	0.24	0.46	0.16	0.06	2.46
PML-NBs	0.51	11.76	0.35	7.69	0.30	0.31	0.33	0.81	0.36	0.12	2.95
Chromatin	0.38	9.45	0.29	6.33	0.34	0.36	0.37	0.86	0.39	0.17	3.27
Perinucleolar	0.58	14.42	0.38	7.88	0.34	0.37	0.42	0.79	0.46	0.08	3.09
Nuclear Speckle	0.30	7.72	0.23	5.60	0.35	0.38	0.45	0.77	0.24	0.07	3.11

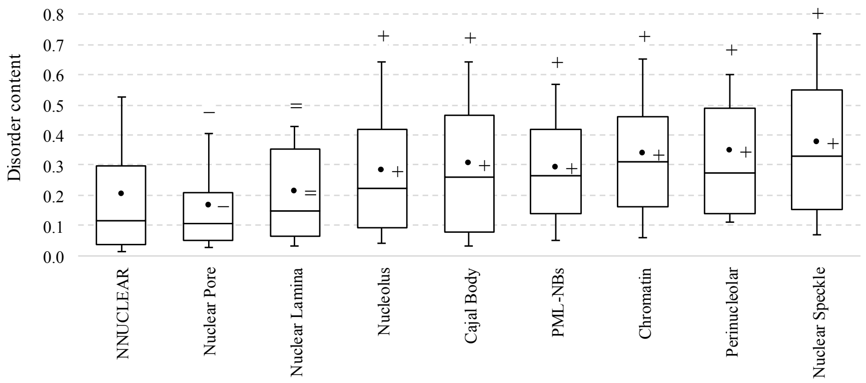

Figure S2. Disorder content of the non-nuclear proteins from the NNUCLEAR dataset and proteins in the considered intra-nuclear compartment from the NUCLEAR*a* dataset. The box plots include the first quartile, median and third quartile while whiskers correspond to the 10th and 90th centiles of the disorder content of proteins in a given protein set. The black circle marker is the mean value of the disorder content. The significance of the differences in the median (mean) disorder content between proteins in a given compartment and non-nuclear proteins is annotated above the whiskers (right ofthe marker); + and − mean that the content of the nuclear proteins is significantly higher and lower (p-value < 0.01), respectively; "=" means that the difference is not significant (p-value ≥ 0.01). Disorder was annotated with the consensus of Espritz and IUPred.

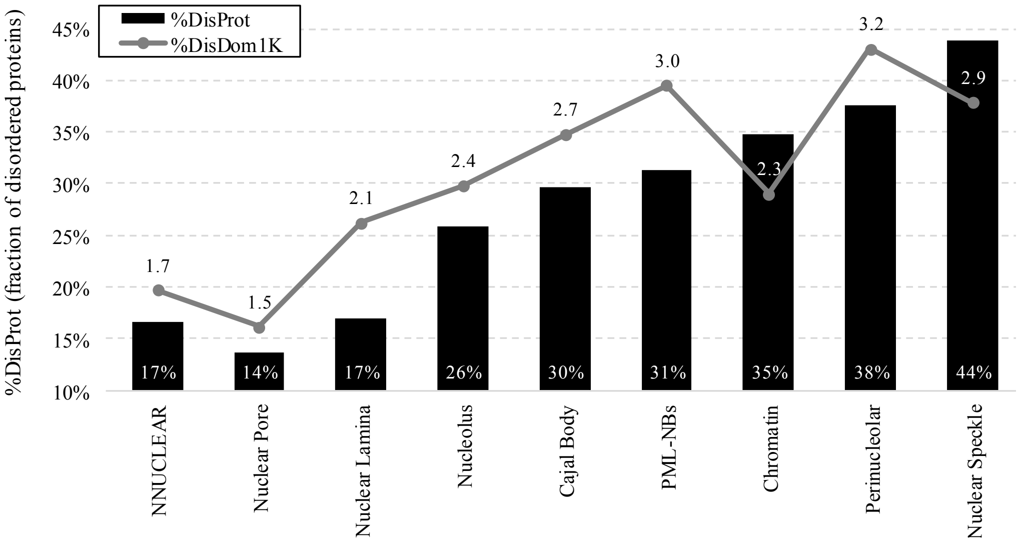

Figure S3. Fraction of disordered proteins (black bars) and normalized number of disordered domains (gray line) for the non-nuclear proteins from the NNUCLEAR dataset and proteins in the considered intra-nuclear compartment from the NUCLEAR*a* dataset. Disorder was annotated with the consensus of Espritz and IUPred.

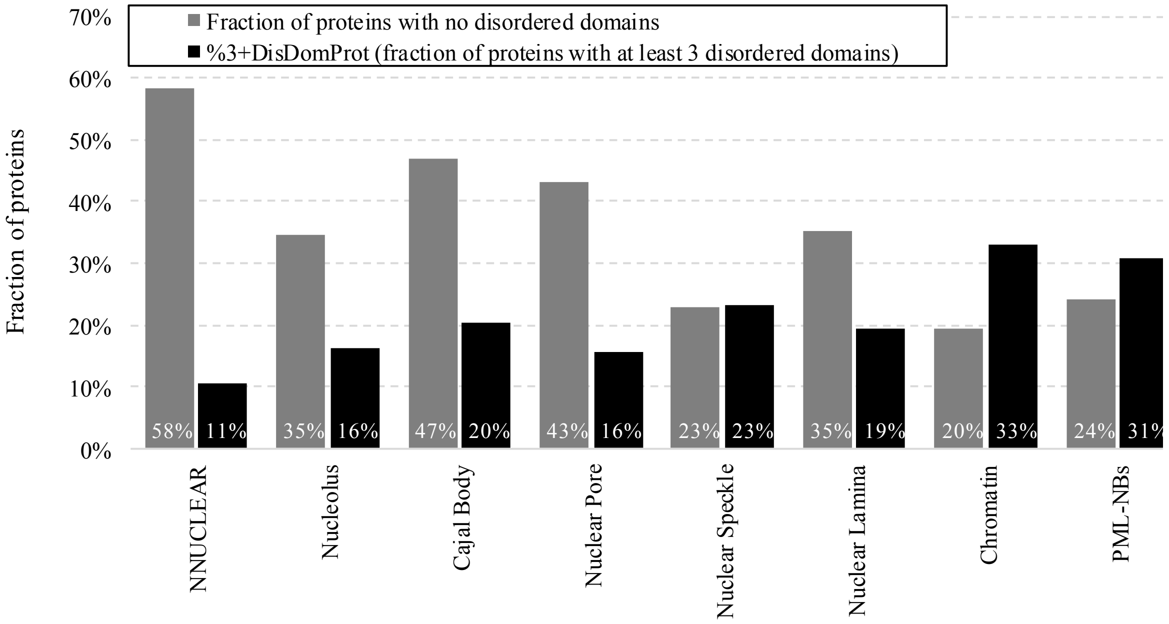

Figure S4. Fraction of proteins with no disordered domains (gray bars) and with at least 3 disordered domains (%3+DisDomProt) (black bars) for the non-nuclear proteins from the NNUCLEAR dataset and proteins in the considered intra-nuclear compartment from the NUCLEAR$_a$ dataset. Disorder was annotated with the consensus of Espritz and IUPred.

Table S3. Disorder content, fraction of disordered proteins, and abundance of disordered domains for the nuclear proteins from the NUCLEAR*ap* dataset grouped by the number of intra-nuclear compartments they are localized in. Disorder was annotated with the consensus of Espritz and IUPred.

Number of Compartments	# Proteins	Disorder Content		Fraction of Disordered Proteins %DisProt	Fraction of Proteins with Disordered Domains			Normalized Number of Disordered Domains %DisDom1K
		Median	Mean		%DisDomProt	%3+DisDomProt	%5+DisDomProt	
1	2350	0.28	0.32	0.33	0.71	0.21	0.07	2.87
2	567	0.28	0.31	0.30	0.71	0.20	0.06	2.84
3	78	0.28	0.31	0.28	0.73	0.26	0.12	2.57
4	8	0.10	0.12	0.00	0.50	0.00	0.00	0.61
5	1	0.45	0.45	1.00	0.00	0.00	0.00	0.00
6	1	0.08	0.08	0.00	0.00	0.00	0.00	0.00

Table S4. Number of protein-protein interactions (PPIs), disorder content, fraction of disordered proteins, and abundance of disordered domains for the nuclear hub proteins and intra-nuclear hub proteins in specific intra-nuclear compartments from the $PPI_{NUCLEARap}$ datasets and for the non-nuclear hub proteins from the $PPI_{NNUCLEAR}$ dataset. Disorder was annotated with the consensus of Espritz and IUPred.

Localization	Average number of PPIs in Hub Proteins	Disorder Content of Hubs		Average number of PPIs in Intra-Compartment Hub Proteins	Disorder Content of Intra-Compartment Hubs		Fraction of Disordered Proteins %DisProt	Fraction of Proteins with Disordered Domains			Normalized Number of Disordered Domains %DisDom1K
		Median	Mean		Median	Mean		%DisDomProt	%3+DisDomProt	%5+DisDomProt	
Non-nuclear	20.41	0.13	0.21	20.41	0.13	0.21	0.17	0.54	0.15	0.05	1.69
Nuclear Pore	21.00	0.12	0.17	17.82	0.08	0.16	0.13	0.53	0.33	0.20	1.99
Nuclear Lamina	18.16	0.26	0.28	14.93	0.21	0.26	0.32	0.63	0.42	0.26	2.96
Nucleolus	31.20	0.22	0.25	28.19	0.20	0.24	0.21	0.66	0.13	0.02	2.33
Cajal Body	23.87	0.17	0.27	21.82	0.08	0.20	0.27	0.47	0.13	0.00	1.99
PML-NBs	20.10	0.28	0.31	17.31	0.26	0.30	0.32	0.77	0.34	0.10	2.89
Chromatin	20.20	0.35	0.37	16.60	0.33	0.35	0.38	0.87	0.39	0.17	3.33
Perinucleolar	22.43	0.34	0.37	17.33	0.34	0.33	0.43	0.79	0.50	0.07	3.11
Nuclear Speckle	19.74	0.30	0.33	18.20	0.27	0.31	0.38	0.75	0.25	0.07	2.92

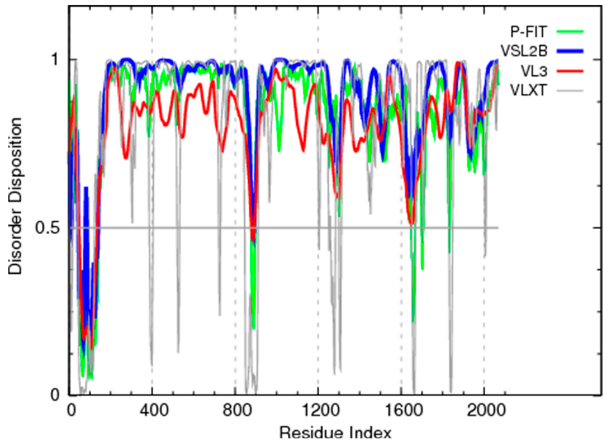

(**A**)

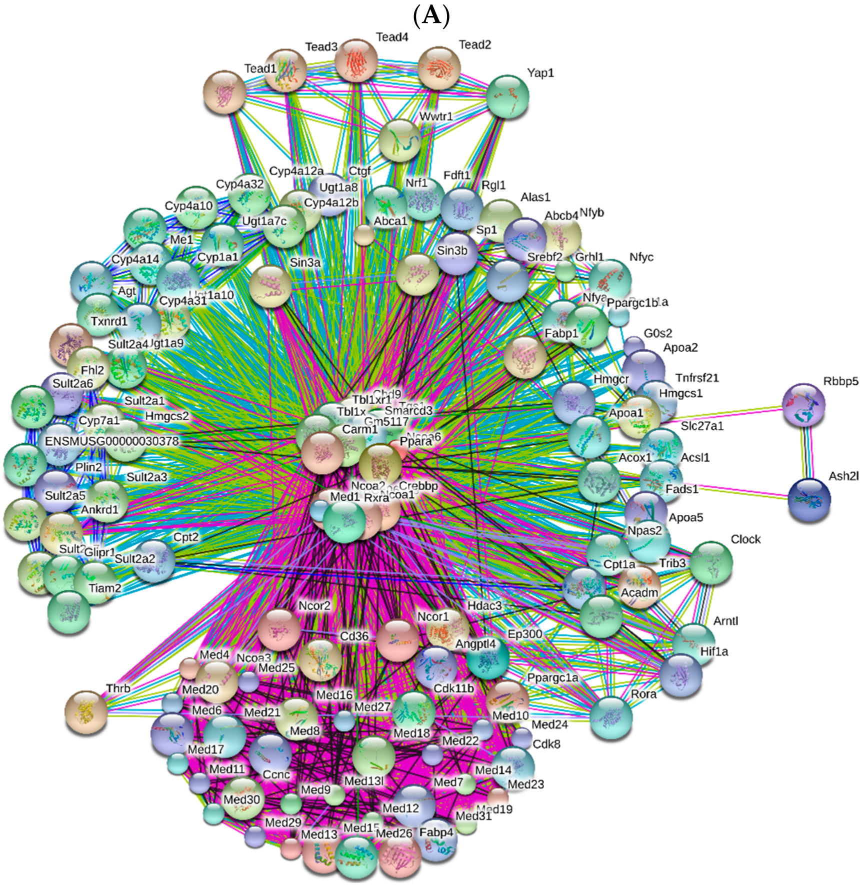

(**B**)

Figure S5. Abundance and functionality of intrinsic disorder in the nuclear receptor coactivator 6 (UniProt ID: Q9JL19). (**A**) Evaluation of the per-residue disorder propensity by the members of the PONDR family of disorder predictors. A disorder threshold is indicated as a thin line (at score of 0.5) to show a boundary between disorder (>0.5) and order (<0.5); (**B**) Analysis of the interactivity of the nuclear pore complex-associated intra-nuclear coiled-coil protein TPR (UniProt ID: Q7M739) by STRING computational platform [76]. STRING produces the network of predicted associations for a particular protein and its interactome. The network nodes are proteins, whereas the edges represent the predicted or known functional associations. There are seven types of evidence used in predicting the associations which are indicated in the resulting network by the differently colored lines, where a red line indicates the presence of fusion evidence; a green line—neighborhood evidence; a blue line—co-occurrence evidence; a purple line—experimental evidence; a yellow line—text mining evidence; a light blue line—database evidence; a black line—co-expression evidence [76].

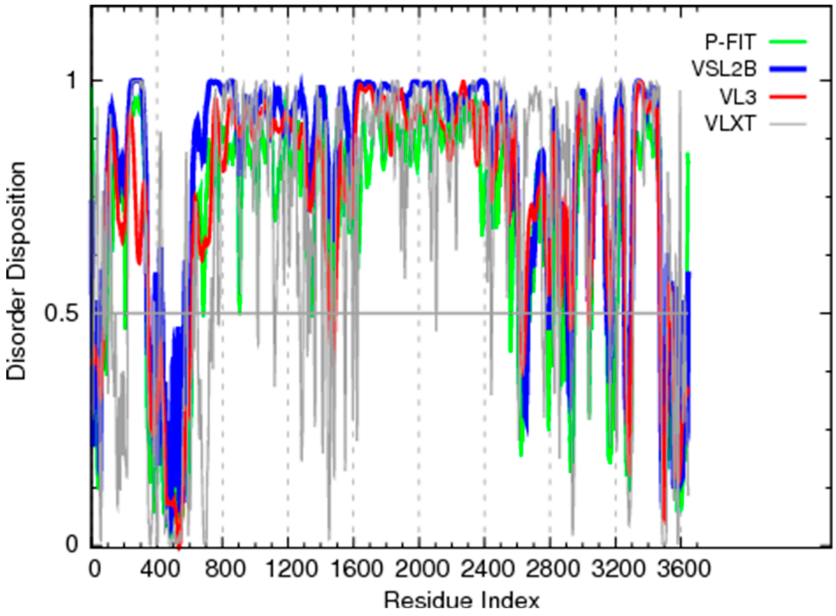

(**A**)

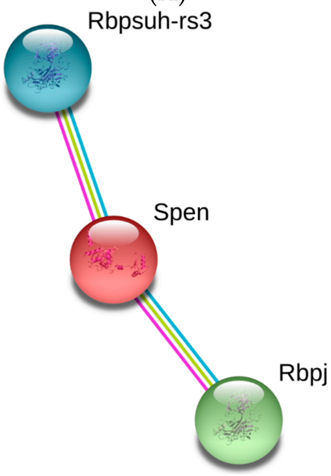

(**B**)

Figure S6. Abundance and functionality of intrinsic disorder in the Msx2-interacting protein (UniProt ID: Q62504). (**A**) Evaluation of the per-residue disorder propensity by the members of the PONDR family of disorder predictors. A disorder threshold is indicated as a thin line (at score of 0.5) to show a boundary between disorder (>0.5) and order (<0.5); (**B**) Analysis of the interactivity of the bloom syndrome protein homolog (UniProt ID: O88700) by STRING computational platform [76]. STRING produces the network of predicted associations for a particular protein and its interactome. The network nodes are proteins, whereas the edges represent the predicted or known functional associations. There are seven types of evidence used in predicting the associations which are indicated in the resulting network by the differently colored lines, where a red line indicates the presence of fusion evidence; a green line—neighborhood evidence; a blue line—co-occurrence evidence; a purple line—experimental evidence; a yellow line—text mining evidence; a light blue line—database evidence; a black line—co-expression evidence [76].

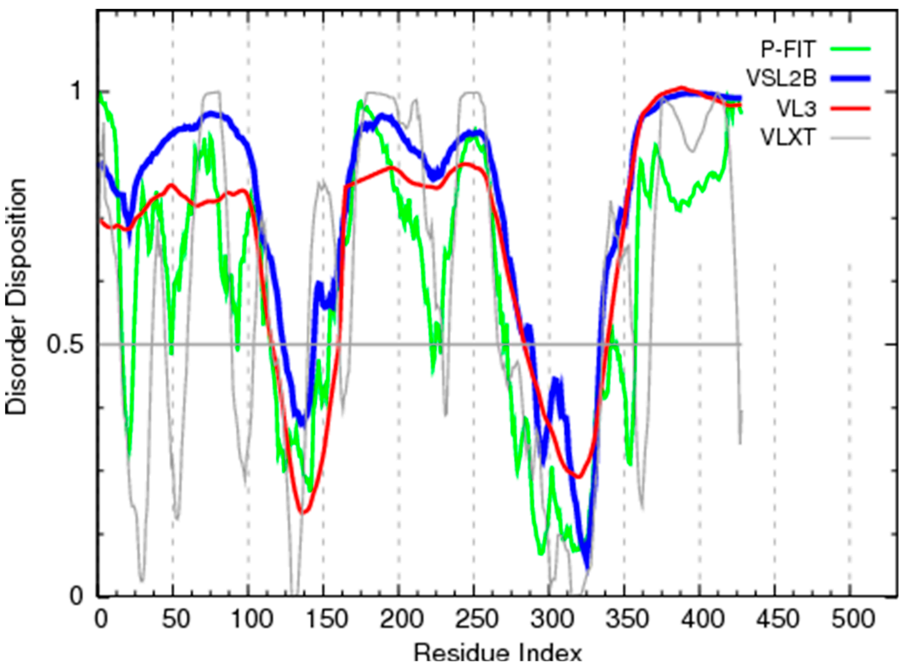

(**A**)

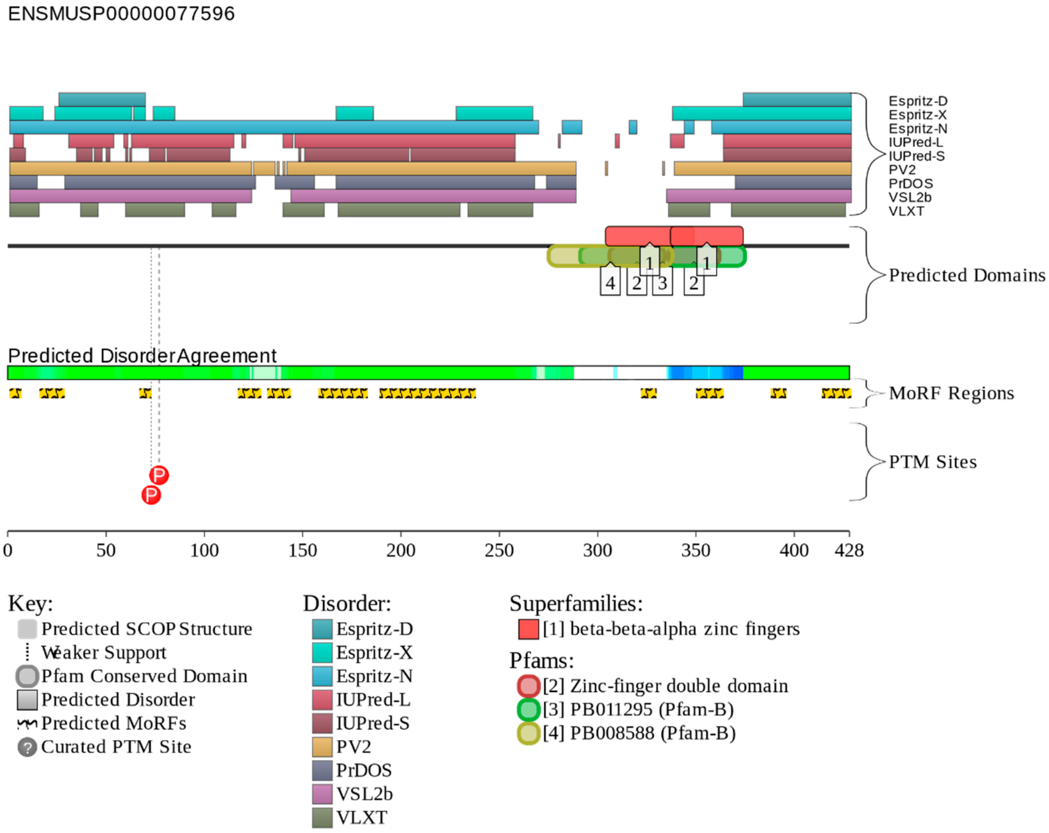

(**B**)

Figure S7. *Cont.*

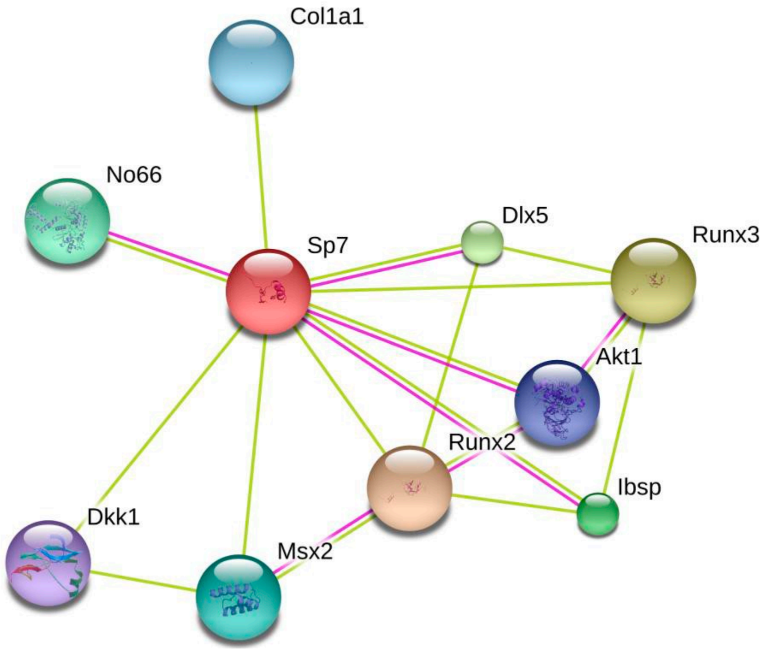

(**C**)

Figure S7. Abundance and functionality of intrinsic disorder in the transcription factor Sp7 (UniProt ID: Q8VI67). (**A**) Evaluation of the per-residue disorder propensity by the members of the PONDR family of disorder predictors. A disorder threshold is indicated as a thin line (at score of 0.5) to show a boundary between disorder (>0.5) and order (<0.5); (**B**) Evaluation of the functional intrinsic disorder propensity by the D^2P^2 database (http://d2p2.pro/) [55]. In the corresponding plot, top nine colored bars represent location of disordered regions predicted by different disorder predictors (Espritz-D, Espritz-N, Espritz-X, IUPred-L, IUPred-S, PV2, PrDOS, PONDR® VSL2b, and PONDR® VLXT, see keys for the corresponding color codes). Green-and-white bar in the middle of the plot shows the predicted disorder agreement between these nine predictors, with green parts corresponding to disordered regions by consensus. Location of predicted and known domains are shown by numbered colored bars, with explanations of numbers being shown in keys below the plot. Yellow bar shows the location of the predicted disorder-based binding site (MoRF region), whereas red circles with "P" inside at the bottom of the plots show locations of phosphorylation sites. Vertical dashed lines show actual positions of the phosphorylation sites; (**C**) Analysis of the interactivity of the bloom syndrome protein homolog (UniProt ID: O88700) by STRING computational platform [76]. STRING produces the network of predicted associations for a particular protein and its interactome. The network nodes are proteins, whereas the edges represent the predicted or known functional associations. There are seven types of evidence used in predicting the associations which are indicated in the resulting network by the differently colored lines, where a red line indicates the presence of fusion evidence; a green line—neighborhood evidence; a blue line—co-occurrence evidence; a purple line—experimental evidence; a yellow line—text mining evidence; a light blue line—database evidence; a black line—co-expression evidence [76].

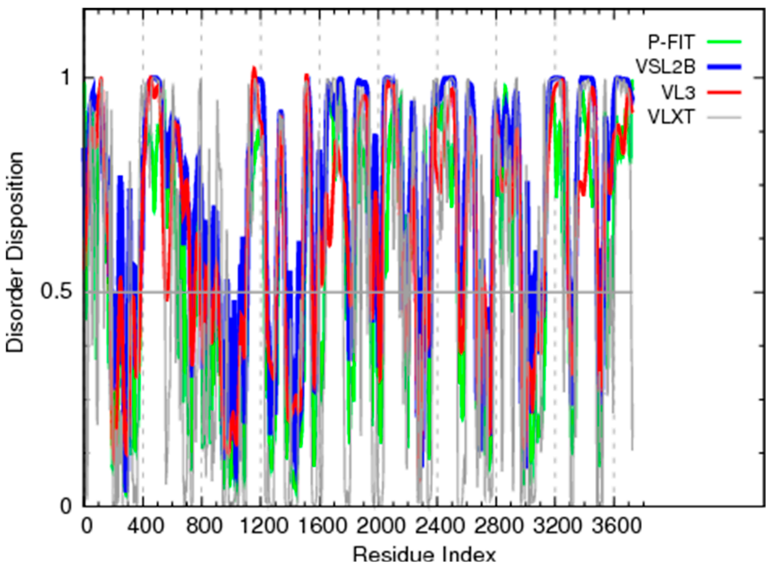

(**A**)

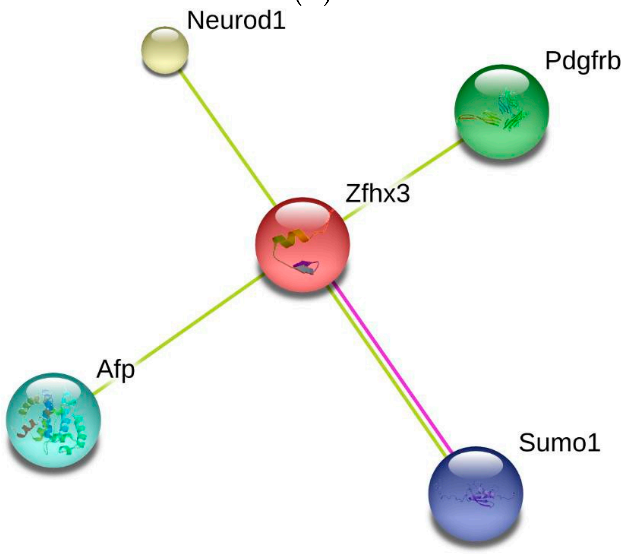

(**B**)

Figure S8. Abundance and functionality of intrinsic disorder in the zinc finger homeobox protein 3 (UniProt ID: Q61329). (**A**) Evaluation of the per-residue disorder propensity by the members of the PONDR family of disorder predictors. A disorder threshold is indicated as a thin line (at score of 0.5) to show a boundary between disorder (>0.5) and order (<0.5); (**B**) Analysis of the interactivity of the bloom syndrome protein homolog (UniProt ID: O88700) by STRING computational platform [76]. STRING produces the network of predicted associations for a particular protein and its interactome. The network nodes are proteins, whereas the edges represent the predicted or known functional associations. There are seven types of evidence used in predicting the associations which are indicated in the resulting network by the differently colored lines, where a red line indicates the presence of fusion evidence; a green line—neighborhood evidence; a blue line—co-occurrence evidence; a purple line—experimental evidence; a yellow line—text mining evidence; a light blue line—database evidence; a black line—co-expression evidence [76].

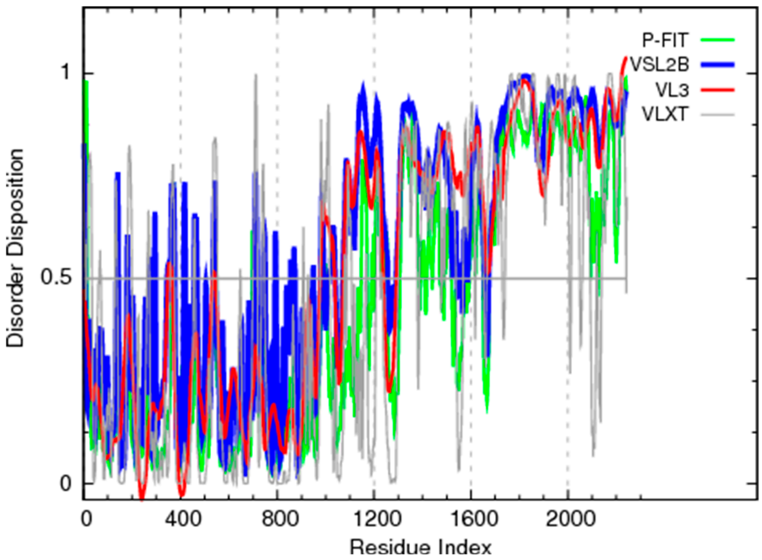

(**A**)

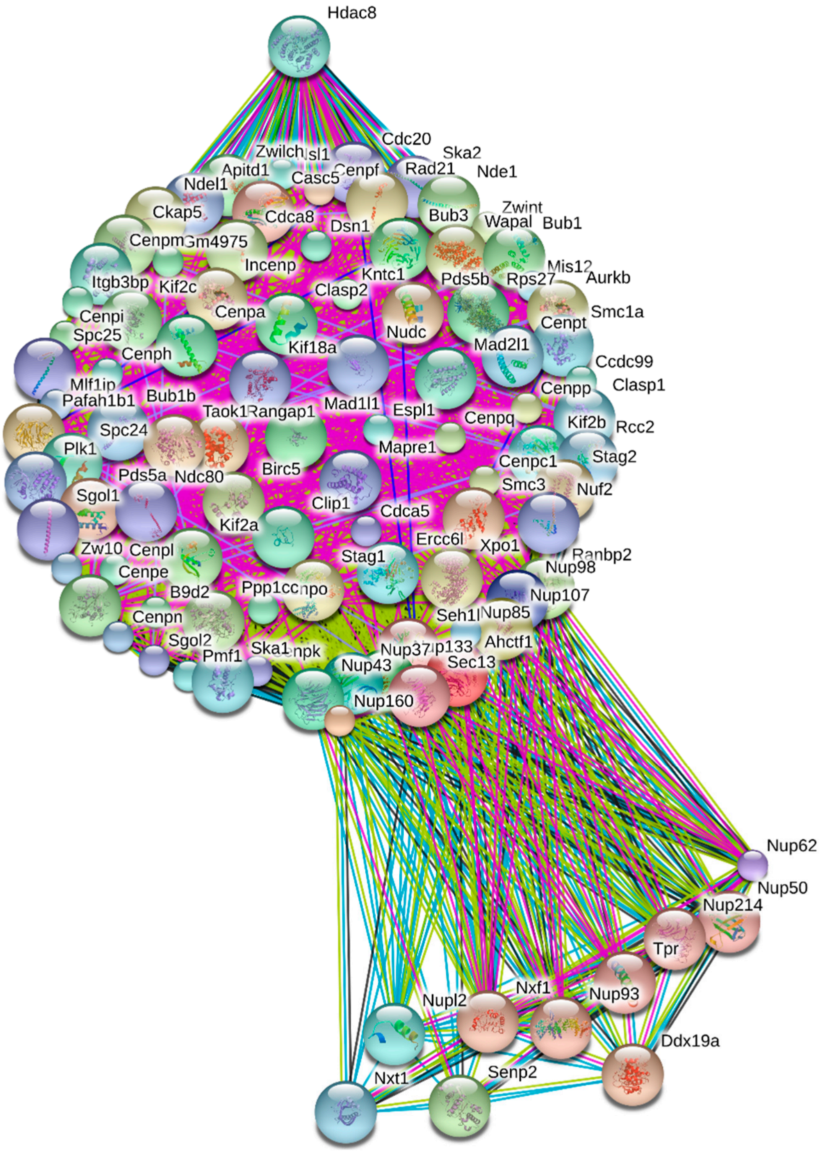

(**B**)

Figure S9. *Cont.*

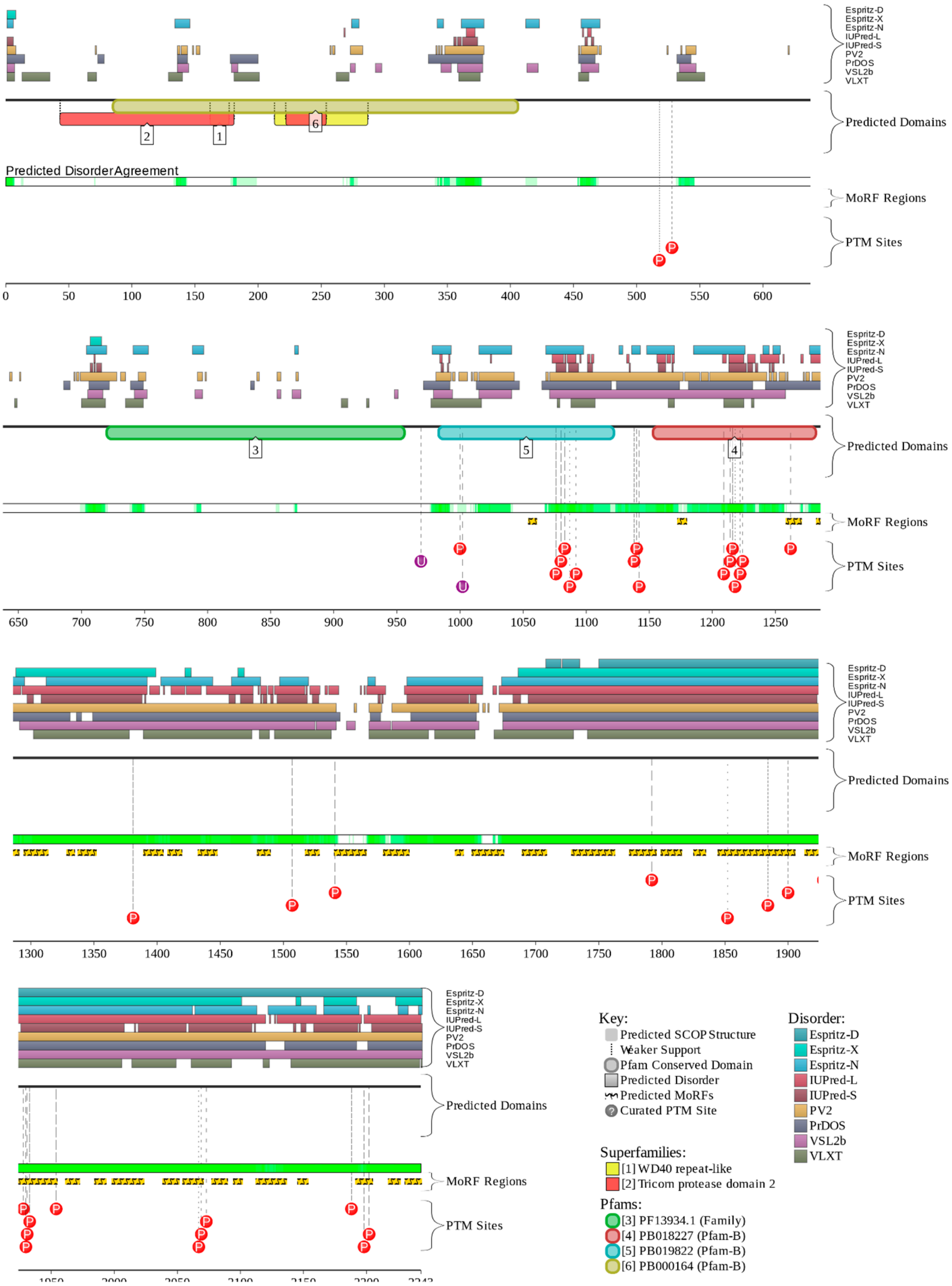

(**C**)

Figure S9. Abundance and functionality of intrinsic disorder in the protein ELYS (UniProt ID: Q8CJF7). (**A**) Evaluation of the per-residue disorder propensity by the members of the PONDR family of disorder predictors. A disorder threshold is indicated as a thin line (at score of 0.5) to show a boundary between disorder (>0.5) and order (<0.5); (**B**) Analysis of the interactivity of the bloom syndrome protein homolog (UniProt ID: O88700) by STRING computational platform [76]. STRING produces the network of predicted associations for a particular protein and its interactome. The network nodes are proteins, whereas the edges represent the predicted or known functional associations. There are seven types of evidence used in predicting the associations which are indicated in the resulting network by the differently colored lines, where a red line indicates the presence of fusion evidence; a green line—neighborhood evidence; a blue line—co-occurrence evidence; a purple line—experimental evidence;

a yellow line—text mining evidence; a light blue line—database evidence; a black line—co-expression evidence [76]; (**C**) Evaluation of the functional intrinsic disorder propensity by the D²P² database (http://d2p2.pro/) [55]. In the corresponding plot, top nine colored bars represent location of disordered regions predicted by different disorder predictors (Espritz-D, Espritz-N, Espritz-X, IUPred-L, IUPred-S, PV2, PrDOS, PONDR® VSL2b, and PONDR® VLXT, see keys for the corresponding color codes). Green-and-white bar in the middle of the plot shows the predicted disorder agreement between these nine predictors, with green parts corresponding to disordered regions by consensus. Yellow bar shows the location of thepredicted disorder-based binding site (MoRF region), whereas red and yellow circles at the bottom of the plots show locations of phosphorylation and acetylation sites, respectively.

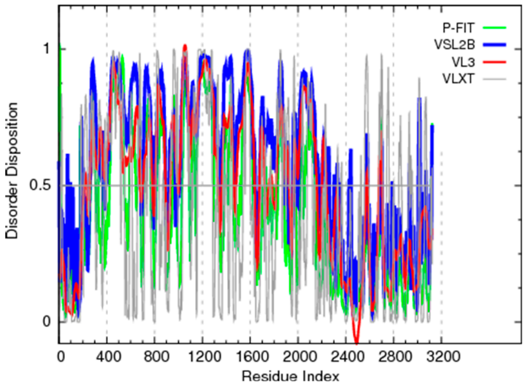

(**A**)

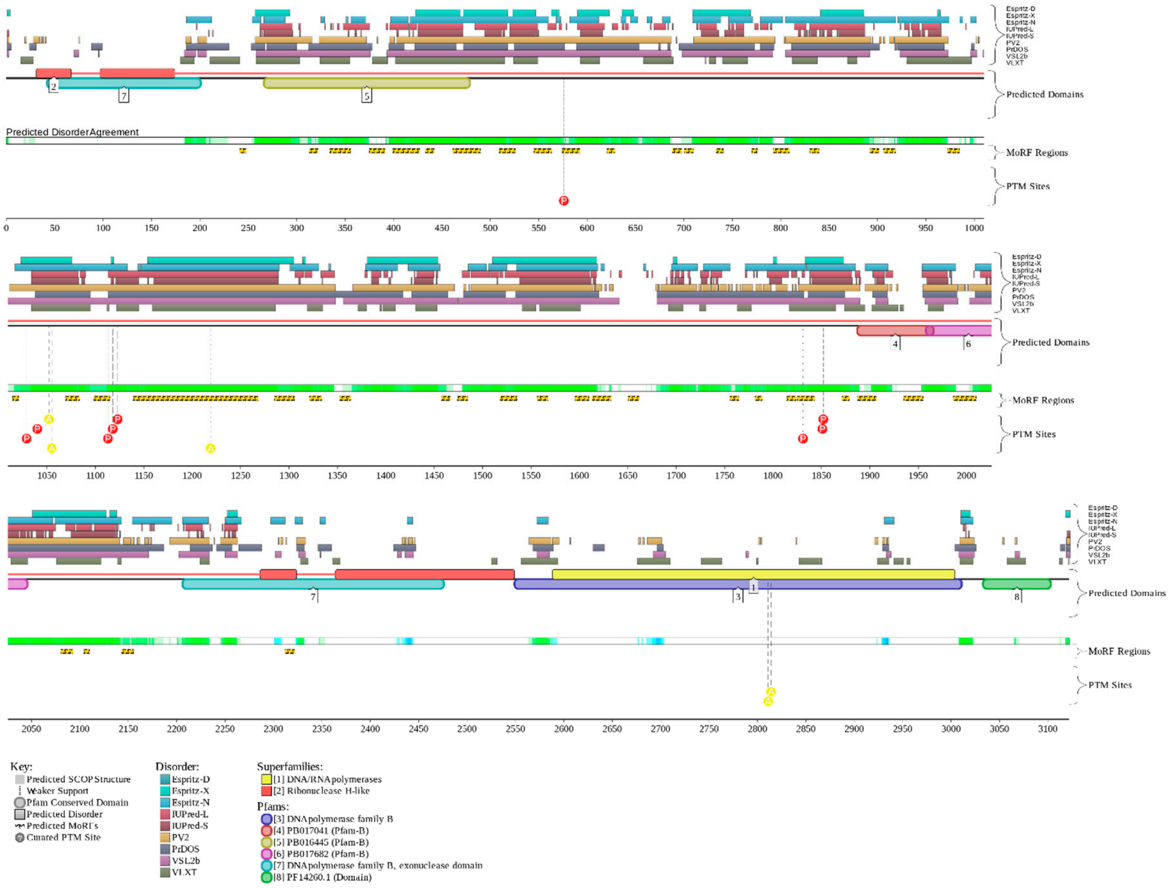

(**B**)

Figure S10. *Cont.*

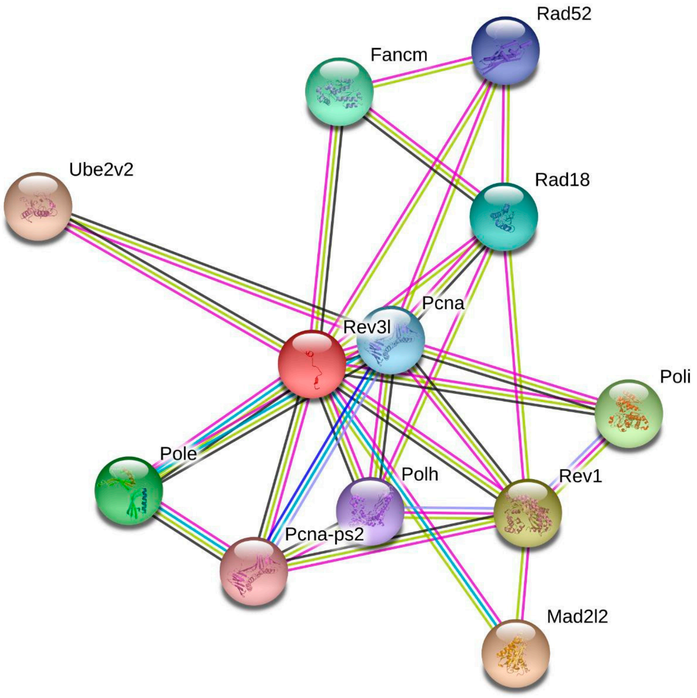

(**C**)

Figure S10. Abundance and functionality of intrinsic disorder in the DNA polymerase zeta catalytic subunit (UniProt ID: Q61493). (**A**) Evaluation of the per-residue disorder propensity by the members of the PONDR family of disorder predictors. A disorder threshold is indicated as a thin line (at score of 0.5) to show a boundary between disorder (>0.5) and order (<0.5); (**B**) Evaluation of the functional intrinsic disorder propensity by the D²P² database (http://d2p2.pro/) [55]. In the corresponding plot, top nine colored bars represent location of disordered regions predicted by different disorder predictors (Espritz-D, Espritz-N, Espritz-X, IUPred-L, IUPred-S, PV2, PrDOS, PONDR® VSL2b, and PONDR® VLXT, see keys for the corresponding color codes). Green-and-white bar in the middle of the plot shows the predicted disorder agreement between these nine predictors, with green parts corresponding to disordered regions by consensus. Yellow bar shows the location of the predicted disorder-based binding site (MoRF region), whereas red and yellow circles at the bottom of the plots show locations of phosphorylation and acetylation sites, respectively; (**C**) Analysis of the interactivity of the bloom syndrome protein homolog (UniProt ID: O88700) by STRING computational platform [76]. STRING produces the network of predicted associations for a particular protein and its interactome. The network nodes are proteins, whereas the edges represent the predicted or known functional associations. There are seven types of evidence used in predicting the associations which are indicated in the resulting network by the differently colored lines, where a red line indicates the presence of fusion evidence; a green line—neighborhood evidence; a blue line—co-occurrence evidence; a purple line—experimental evidence; a yellow line—text mining evidence; a light blue line—database evidence; a black line—co-expression evidence [76].

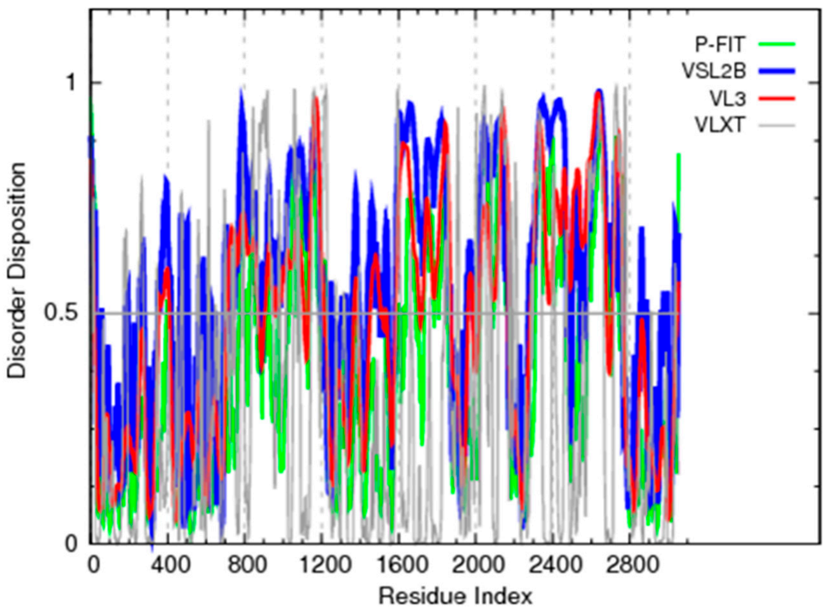

(**A**)

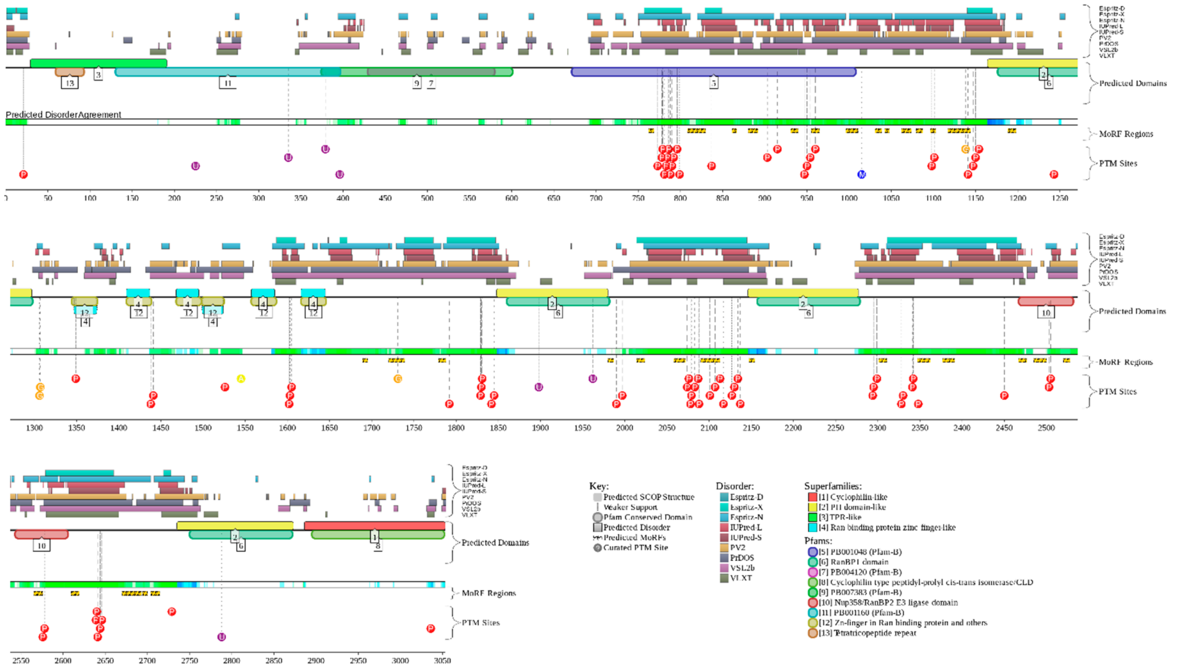

(**B**)

Figure S11. *Cont.*

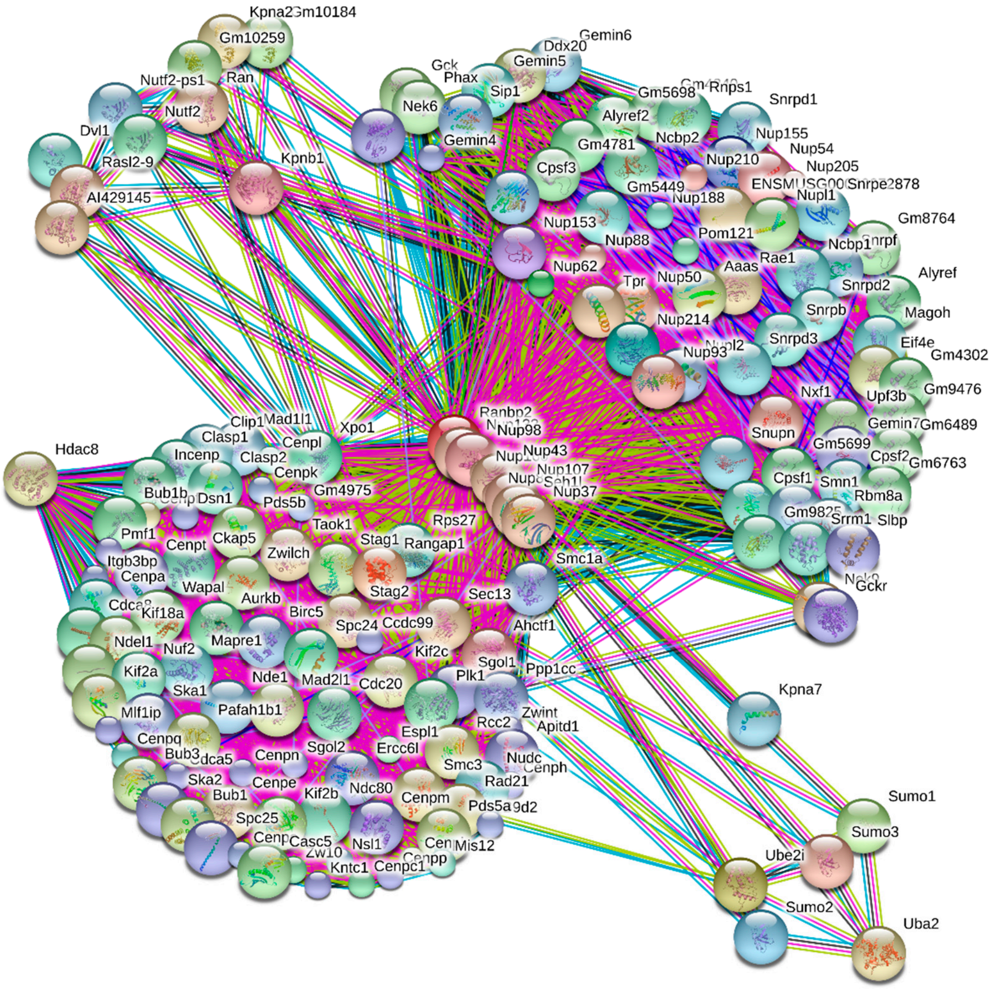

(**C**)

Figure S11. Abundance and functionality of intrinsic disorder in the E3 SUMO-protein ligase RanBP2 (UniProt ID: Q9ERU9). (**A**) Evaluation of the per-residue disorder propensity by the members of the PONDR family of disorder predictors. A disorder threshold is indicated as a thin line (at score of 0.5) to show a boundary between disorder (>0.5) and order (<0.5); (**B**) Evaluation of the functional intrinsic disorder propensity by the D[2]P[2] database (http://d2p2.pro/) [55]. In the corresponding plot, top nine colored bars represent location of disordered regions predicted by different disorder predictors (Espritz-D, Espritz-N, Espritz-X, IUPred-L, IUPred-S, PV2, PrDOS, PONDR® VSL2b, and PONDR® VLXT, see keys for the corresponding color codes). Green-and-white bar in the middle of the plot shows the predicted disorder agreement between these nine predictors, with green parts corresponding to disordered regions by consensus. Yellow bar shows the location of the predicted disorder-based binding site (MoRF region), whereas red and yellow circles at the bottom of the plots show locations of phosphorylation and acetylation sites, respectively; (**C**) Analysis of the interactivity of the bloom syndrome protein homolog (UniProt ID: O88700) by STRING computational platform [76]. STRING produces the network of predicted associations for a particular protein and its interactome. The network nodes are proteins, whereas the edges represent the predicted or known functional associations. There are seven types of evidence used in predicting the associations which are indicated in the resulting network by the differently colored lines, where a red line indicates the presence of fusion evidence; a green line—neighborhood evidence; a blue line—co-occurrence evidence; a purple line—experimental evidence; a yellow line—text mining evidence; a light blue line—database evidence; a black line—co-expression evidence [76].

Table S5. Characterization of several selected mouse nuclear proteins that are co-localized in at least three sub-nuclear compartments and have at least five disordered domains.

Protein	UniProt ID	Protein Length (N_{AIBS}) [a]	Sub-Nuclear Compartments	PONDR-FIT (%) [b]	MobiDB Consensus (%) [c]	Two HT Consensus (%) [d]	Location (Length) of Disordered Domains [e]	Location (Length) of AIBSs [f]
Nuclear receptor coactivator 6	Q9JL19	2067 (27/13.1/18.7)	Chromatin Nuclear Speckle PML-NBs	92.8	84.91	84.7	175–715 (541)<br>719–1282 (564)<br>1288–1413 (126)<br>1417–1580 (164)<br>1709–1822 (114)<br>1839–1912 (74)<br>1934–2066 (133)	159–261 (103); 304–374 (71); 378–614 (237); 621–840 (220); 842–865 (24); 872–909 (38); 926–1002 (77); 1030–1210 (181); 1217–1226 (10); 1239–1320 (82); 1351–1461 (111); 1467–1539 (73); 1556–1575 (20); 1598–1606 (9); 1622–1633 (12); 1658–1672 (15); 1696–1713 (18); 1730–1770 (41); 1791–1804 (14); 1822–1843 (22); 1859–1877 (19); 1886–1897 (12); 1904–1933 (30); 1943–1974 (32); 1979–2017 (39); 2029–2036 (8); 2044–2065 (22);
Nuclear pore complex-associated intra-nuclear coiled-coil protein TPR	Q7M739	2357 (29/12.3/37.4)	Nuclear Pore Nuclear Speckle Nucleolus	82.7	N/D [g]	40.0	909–954 (46)<br>1451–1517 (67)<br>1602–1757 (156)<br>1765–2133 (369)<br>2153–2182 (30)<br>2218–2356 (139)	219–226 (8); 254–260 (7); 275–292 (18); 307–315 (9); 500–505 (6); 611–639 (29); 670–682 (13); 718–728 (11); 815–821 (7); 884–916 (33); 962–973 (12); 1084–1096 (13); 1298–1308 (11); 1418–1424 (7); 1440–1445 (6); 1550–1567 (18); 1591–1613 (23); 1637–1703 (67); 1711–1745 (35); 1752–1775 (24); 1788–1802 (15); 1804–1823 (20); 1834–2064 (231); 2071–2128 (58); 2130–2162 (33); 2173–2199 (27); 2206–2282 (77); 2286–2296 (11); 2305–2357 (53);

Table S5. *Cont.*

Protein	UniProt ID	Protein Length (N_{AIBS}) [a]	Sub-Nuclear Compartments	PONDR-FIT (%) [b]	MobiDB Consensus (%) [c]	Two HT Consensus (%) [d]	Location (Length) of Disordered Domains [e]	Location (Length) of AIBSs [f]
Msx2-interacting protein	Q62504	3644 (77/21.1/51.1)	Chromatin Nuclear Speckle Perinucleolar Compartment	76.6	73.74	71.1	99–330 (232) 698–900 (203) 904–1418 (515) 1508–1538 (31) 1545–2472 (928) 2475–2531 (57) 2744–2780 (37) 2951–3027 (77) 3083–3134 (52) 3278–3474 (197)	48–58 (11); 89–117 (29); 143–152 (10); 158–171 (14); 176–189 (14); 206–228 (23); 236–242 (7); 252–265 (14); 278–288 (11); 308–326 (19); 337–343 (7); 594–599 (6); 650–657 (8); 680–699 (20); 712–721 (10); 776–809 (34); 890–915 (26); 917–942 (26); 953–968 (16); 976–984 (9); 986–1011 (26); 1029–1034 (6); 1067–1111 (45); 1120–1138 (19); 1144–1152 (9); 1169–1188 (20); 1196–1228 (33); 1246–1263 (18); 1276–1284 (9); 1294–1302 (9); 1309–1329 (21); 1336–1348 (13); 1365–1381 (17); 1412–1423 (12); 1444–1452 (9); 1507–1512 (6); 1540–1554 (15); 1571–1584 (14); 1589–1609 (21); 1647–1788 (142); 1804–1834 (31); 1842–1860 (19); 1864–1871 (8); 1877–1900 (24); 1911–1934 (24); 1946–1964 (19); 1996–2018 (23); 2020–2038 (19); 2065–2131 (67); 2138–2301 (164); 2308–2325 (18); 2343–2359 (17); 2366–2377 (12); 2393–2456 (64); 2467–2486 (20); 2496–2503 (8); 2521–2568 (48); 2724–2730 (7); 2760–2769 (10); 2780–2796 (17); 2807–2827 (21); 2851–2871 (21); 2916–2931 (16); 2933–2955 (23); 2964–2976 (13); 2984–2994 (11); 3008–3018 (11); 3022–3076 (55); 3078–3092 (15); 3120–3126 (7); 3136–3179 (44); 3204–3216 (13); 3241–3251 (11); 3260–3314 (55); 3335–3458 (124); 3477–3497 (21); 3553–3558 (6);

Table S5. *Cont.*

Protein	UniProt ID	Protein Length (NAIBS) [a]	Sub-Nuclear Compartments	PONDR-FIT (%) [b]	MobiDB Consensus (%) [c]	Two HT Consensus (%) [d]	Location (Length) of Disordered Domains [e]	Location (Length) of AIBSs [f]
Transcription factor Sp7	Q8VI67	428 (11/25.7/39.3)	Chromatin Nucleolus Nuclear Speckle	68.5	67.06	61.9	25–68 (44) 71–111 (41) 150–202 (53) 204–256 (53) 363–427 (65)	1–6 (6); 16–28 (13); 67–72 (6); 117–128 (12); 132–143 (12); 158–182 (25); 189–237 (49); 322–329 (8); 350–363 (14); 388–395 (8); 414–428 (15);
Zinc finger homeobox protein 3	Q61329	3726 (70/18.8/39.3)	Nuclear Pore Nuclear Speckle Nucleolus	59.0	52.76	48.3	0–80 (81) 92–131 (40) 410–559 (150) 570–622 (53) 1111–1227 (117) 1314–1360 (47) 1493–1540 (48) 1627–1749 (123) 1864–1949 (86) 2032–2091 (60) 2209–2248 (40) 2358–2534 (177) 2602–2656 (55) 2847–2883 (37) 2919–2959 (41) 3133–3276 (144) 3374–3477 (104) 3571–3725 (155)	1–34 (34); 47–66 (20); 77–99 (23); 126–151 (26); 363–370 (8); 393–427 (35); 435–466 ((32); 489–511 (23); 517–549 (33); 555–594 (40); 622–631 (10); 769–787 (19); 807–812 (6); 1100–1105 (6); 1135–1145 (11); 1164–1174 (11); 1186–1198 ((13); 1231–1245 (15); 1261–1269 (9); 1274–1283 (10); 1293–1299 (7); 1305–1316 (12); 1473–1486 (14); 1541–1550 ((10); 1563–1586 (24); 1608–1642 (35); 1654–1659 (6); 1670–1716 (47); 1721–1732 (12); 1776–1783 (8); 1801–1810 (10); 1864–1877 (14); 1883–1891 (9); 1914–1945 (32); 1948–1967 (20); 1990–2008 (19); 2014–2039 (26); 2068–2075 (8); 2088–2121 (34); 2130–2147 (18); 2163–2174 (12); 2192–2205 (14); 2258–2270 (13); 2286–2298 (13); 2377–2386 (10); 2393–2443 (51); 2460–2491 (32); 2508–2523 (16); 2529–2550 (22); 2574–2605 (32); 2622–2630 (9); 2659–2672 (14); 2689–2697 (9); 2723–2731 (9); 2755–2763 (9); 2807–2851 (45); 2882–2915 (34); 2962–2974 (13); 3112–3202 (91); 3218–3261 (44); 3273–3299 (27); 3306–3319 (14); 3340–3364 (25); 3427–3440 (14); 3452–3461 (10); 3475–3494 (20); 3567–3582 (16); 3591–3603 (13); 3634–3699 (66); 3710–3726 (17);

Table S5. *Cont.*

Protein	UniProt ID	Protein Length (N_{AIBS}) [a]	Sub-Nuclear Compartments	PONDR-FIT (%) [b]	MobiDB Consensus (%) [c]	Two HT Consensus (%) [d]	Location (Length) of Disordered Domains [e]	Location (Length) of AIBSs [f]
Protein ELYS	Q8CJF7	2243 (35/15.6/27.0)	Nuclear Lamina Nuclear Pore Nucleolus	43.2	40.12	34.9	1307–1390 (84) 1597–1645 (49) 1681–2111 (431) 2128–2193 (66) 2203–2242 (40)	1054–1060 (7); 1172–1179 (8); 1258–1270 (13); 1282–1290 (9); 1294–1313 (20); 1328–1334 (7); 1337–1351 (15) ; 1389–1404 (16); 1408–1419 (12); 1432–1447 (16) ; 1479–1489 (11) ; 1517–1528 (12); 1540–1565 (26); 1579–1599 (21); 1636–1642 (7); 1649–1674 (26); 1689–1708 (20); 1728–1762 (35); 1774–1795 (22); 1799–1815 (17); 1825–1834 (10); 1844–1905 (62); 1913–1954 (42); 1961–1972 (12); 1984–1993 (10); 1998–2023 (26); 2038–2051 (14); 2054–2070 (17) ; 2077–2089 (13); 2094–2101 (8); 2112–2136 (25); 2145–2153 (9); 2191–2204 (14); 2217–2226 (10); 2230–2243 (14)
Bloom syndrome protein homolog	O88700	1416 (22/15.5/19.4)	Chromatin Nucleolus PML–NBs	38.5	34.96	31.0	0–34 (35) 168–237 (70) 245–292 (48) 443–481 (39) 1294–1361 (68)	1–9 (9); 32–37 (6); 86–93 (8); 131–142 (12); 157–170 (14); 207–217 (11); 232–249 (18); 262–269 (6); 282–329 (48); 376–388 (13); 426–432 (7); 459–466 (8); 488–509 (22); 527–532 (6); 554–564 (11); 610–619 (10); 128,101,291 (11); 1320–1328 (9); 1350–1359 (10); 1368–1381 (14); 1391–1399 (9); 1407–1416(10)

Table S5. *Cont.*

Protein	UniProt ID	Protein Length (NAIBS) [a]	Sub-Nuclear Compartments	PONDR-FIT (%) [b]	MobiDB Consensus (%) [c]	Two HT Consensus (%) [d]	Location (Length) of Disordered Domains [e]	Location (Length) of AIBSs [f]
DNA polymerase zeta catalytic subunit	Q61493	3122 (46/14.7/24.1)	Nuclear Pore Nucleolus PML–NBs	34.8	36.93	27.6	424–470 (47) 483–515 (33) 839–885 (47) 1033–1080 (48) 1153–1287 (135) 1534–1616 (83) 1837–1879 (43) 2084–2135 (52)	241–247 (7); 313–321 (9); 334–355 (22); 375–390 (16); 399–426 (28); 433–441 (9); 461–489 (29); 509–525 (17); 545–563 (19); 574–592 (19); 620–628 (9); 688–697(10); 700–709 (10); 733–740 (8); 770–775 (6); 792–808 (17); 830–839 (10); 892–901 (10); 906–918 (13); 972–984 (13); 1014–1020 (7); 1069–1083 (15); 1098–1114 (17); 1139–1267 (129); 1285–1305 (21); 1321–1333 (13); 1352–1363 (12); 1457–1466 (10); 1474–1484 (11); 1518–1535 (18); 1556–1567 (12); 1595–1609 (15); 1613–1632 (20); 1650–1661 (12); 1755–1764 (10); 1781–1788 (8); 1814–1823 (10); 1825–1842 (18); 1871–1878 (8); 1887–1906 (20); 1935–1954 (20); 1986–2009 (24); 2080–2092 (13); 2104–2109 (6); 2143–2155 (13); 2312–2321 (10)
E3 SUMO-protein ligase RanBP2	Q9ERU9	3053 (33/10.8/12.7)	Chromatin Nuclear Lamina Nuclear Pore	30.2	29.68	21.0	828–863 (36) 1137–1173 (37) 1738–1771 (34) 2027–2066 (40) 2114–2144 (31) 2311–2347 (37) 2393–2435 (43) 2604–2665 (62)	762–768 (7); 808–829 (22); 861–866 (6); 880–891 (12); 931–939 (9); 955–964 (10); 996–1010 (15); 1031–1038 (8); 1042–1047 (6); 1062–1073 (12); 1079–1086 (8); 1096–1102 (7); 1117–1143 (27); 1188–1197 (10); 1689–1694 (6); 1720–1738 (19); 1779–1787 (9); 1980–1986 (7); 2014–2023 (10); 2058–2071 (14); 2090–2112 (23); 2147–2153 (7); 2301–2310 (10); 2347–2361 (15); 2377–2390 (14); 2467–2476 (10); 2485–2499 (15); 2520–2527 (8); 2565–2575 (11); 2609–2618 (19); 2670–2692 (23); 2694–2699 (6); 2704–2714 (11)

[a] NAIBS (A/B/C) represents the number of potential disorder-based binding sites identified by the ANCHOR algorithm (A); the number of AIBSs per 1000 residues of a query protein (B); and the percentage of residues involved in disorder-based interactions (C); [b] Content of disordered residues (*i.e.*, residues with the disorder propensity ≥0.5) in a protein based on the PONDR-FIT disorder prediction [70]; [c] Content of predicted disordered residues in a protein based on the MobiDB consensus score [74,75]; [d] Content of predicted disordered residues in a protein based on a majority vote consensus of two high-throughput predictors, Espritz [68] and IUPred [69]; [e] Information on disordered domain was obtained based on a majority vote consensus of two high-throughput predictors; [f] AIBSs are potential disorder-based binding sites identified by the ANCHOR algorithm [72,73]; [g] Information on Q7M739 is absent in MobiDB.
